# Sleep Deprivation in Mice: Looking Beyond the Slow Wave Rebound

**DOI:** 10.1111/jsr.70232

**Published:** 2025-11-08

**Authors:** Tárek Zoltán Magyar, Orsolya Szalárdy, Róbert Bódizs

**Affiliations:** ^1^ Psychophysiology and Chronobiology Research Group Institute of Behavioural Sciences, Semmelweis University Budapest Hungary; ^2^ Clinical Sleep Disorders Research Group Institute of Psychology, Pázmány Péter Catholic University Budapest Hungary; ^3^ Sound and Speech Perception Research Group Research Centre for Natural Sciences Institute of Cognitive Neuroscience and Psychology Budapest Hungary

**Keywords:** criticality, electrocorticography, normalised spectral entropy, sleep deprivation, slow wave activity, spectral slope

## Abstract

Sleep is a fundamental process supporting the dynamic regulation of neural function. Emerging methods have proposed that the aperiodic components of brain signals (such as the spectral slope, spectral intercept, and spectral knee), in addition to entropy‐based measures, offer robust empirical markers of neural states. The present study investigates the sensitivity of these broadband spectral metrics in comparison to classical band‐limited measures, specifically slow wave activity (SWA; 0.75–4.5 Hz), in a 9‐day mouse sleep deprivation paradigm involving baseline, sleep restriction, and recovery phases (open‐source database). Spectral parameters were computed using the FOOOF algorithm. Results indicate that SWA differentiates between baseline and rebound sleep only during NREM episodes. In contrast, both the spectral slope and spectral intercept capture sleep deprivation‐related changes during both REM and NREM sleep, suggesting these fractal measures reflect sleep homeostasis across stages. Given the shift of the spectral knee towards higher frequencies in mice (~8–10 Hz) as compared to humans (generally around 1 Hz), eliminating the overlap of the spectral slope with the traditional SWA range in these rodents, homeostatic regulation appears to be not strictly bounded to the lower frequencies (0.75–4.5 Hz). Normalised spectral entropy did not differentiate between baseline and recovery sleep, potentially due to its sample size sensitivity. These findings support the empirical utility of broadband spectral parameters in assessing sleep–wake dynamics and highlight their potential to complement or surpass traditional band‐limited metrics.

## 1 Introduction

Sleep is a complex physiological phenomenon with vital functions for brain restoration and cognitive functioning. One well‐established aspect of sleep regulation is its homeostatic component, which manifests as increased electroencephalographic (EEG) slow wave activity (SWA; 0.75–4.5 Hz) following prolonged wakefulness, a response widely interpreted as a compensatory mechanism reflecting sleep pressure (Borbély 1982; Borbély et al. 2016). Traditionally, spectral analysis of neurophysiological signals in this context has focused on band‐limited power estimates within specific frequency ranges, particularly the SWA band during non‐rapid eye movement (NREM) sleep. However, these classical approaches often face limitations such as poor statistical comparability across individuals and the inability to account for underlying structural properties of the EEG signal (Bódizs et al. 2021).

Recent developments in signal processing have proposed a complementary perspective by decomposing the EEG power spectrum into periodic (oscillatory) and aperiodic (non‐oscillatory) components (Donoghue et al. 2020). The aperiodic component, often modelled as a 1/*f*‐like decay in power across frequency, captures the broadband characteristics of neural activity. A key parameter of this decomposition—the spectral slope—reflects the rate of decay in the aperiodic signal and provides a more general measure of brain state than narrowband power alone. Importantly, spectral slope is sensitive to age (Bódizs et al. 2021), arousal (Lendner et al. 2020), cognitive load (Höhn et al. 2024), sleep depth (Horváth et al. 2022) and sleep homeostasis (Horváth and Bódizs 2025), making it a promising index of neural dynamics during sleep. Empirical evidence suggests that steeper spectral slopes are associated with deeper sleep stages and increased homeostatic sleep pressure, showing consistent correlations with classical indicators of high SWA such as early‐night NREM cycles, anterior scalp regions, and younger age (Bódizs et al. 2024). Unlike conventional power metrics that rely on arbitrarily defined frequency bands, spectral slope captures broadband dynamics, offering a more robust and continuous index of cortical excitability and inhibition balance (Gao et al. 2017). Although our study does not directly test critical states, the concept of criticality provides a compelling theoretical framework for understanding large‐scale neural dynamics and optimal brain function (Beggs and Plenz 2003; Chialvo 2010). In this view, the brain operates near a critical point between order and disorder, a regime that maximises computational capacities such as information transmission, adaptability, and dynamic range (Shew and Plenz 2013; Zimmern 2020). Importantly, spectral slope, as a measure of broadband aperiodic neural activity, has been increasingly recognised as a potential empirical marker of such dynamics, capturing 1/*f*‐like power‐law scaling associated with scale invariance (He 2014). Theoretical models suggest that extended wakefulness may gradually shift neural networks towards a supercritical state, characterised by increased excitability and reduced computational efficiency, which is then recalibrated through sleep‐dependent processes (Meisel et al. 2013; Pearlmutter and Houghton 2009). Experimental evidence in animal models further supports this idea, showing that sleep restores markers of balanced, near‐critical activity following wake‐induced deviations (Xu et al. 2024).

Finally, given that brain signals are inherently complex and exhibit scale‐invariant properties, entropy‐based measures have also been explored as indicators of sleep depth and quality (Keshmiri 2020; Touil et al. 2022). While these measures highlight the nonlinear dynamics of brain activity, spectral slope offers a practical and computationally tractable approach to characterising the broadband structure of neurophysiological signals during sleep and wake states. Therefore, the present study investigates the fractal parameters of the neurophysiological power spectrum (spectral slope and spectral intercept) as a broadband index derived from the aperiodic component of the EEG power spectrum, and spectral entropy as a complementary index, reflecting the irregularity and distributional complexity of power across the frequency spectrum. While spectral slope primarily characterises the shape of the power spectrum, spectral entropy quantifies the unpredictability or disorder in the spectral distribution of the signal, and it has previously been associated with arousal level, sleep stage transitions, and depth of sleep (Hou et al. 2018; Touil et al. 2022). Together, these two approaches offer a data‐driven, broadband‐sensitive framework for assessing neural dynamics during sleep and their modulation by extended wakefulness.

## 2 Hypotheses

We studied the sleep deprivation‐related alterations of the ECoG power spectrum of mice exposed to a sleep deprivation paradigm containing baseline, sleep deprivation and sleep recovery phases (open‐source database; Han et al. 2022); experimental days are made up of sleep deprivation (00:00–18:00) and rebound periods (18:00–24:00). Based on the above detailed theoretical background, the following hypotheses were formulated:

*Recovery sleep following deprivation will be reflected by* a *steeper spectral slope and higher spectral intercept compared to baseline levels (pre‐sleep deprivation levels at corresponding times of day)*.

*Slow wave activity will reflect the sleep pressure caused by sleep deprivation by increased values*.

*Normalised spectral entropy will report decreased complexity during recovery sleep*.

## 3 Methods

### 3.1 Sample, Experimental Conditions and Electrocorticograph (ECoG) Recording

**FIGURE 1:**
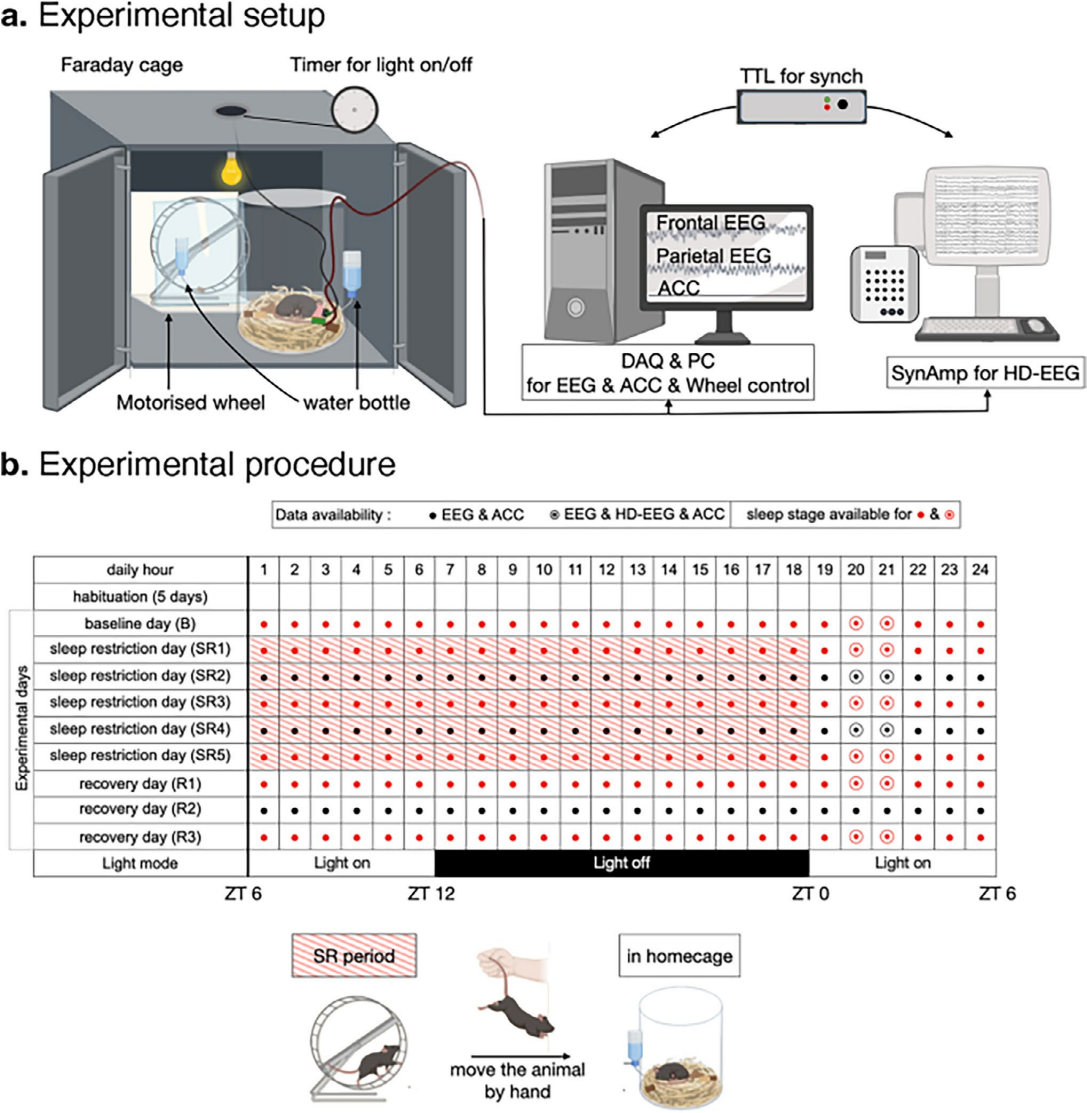
Depiction of the experimental design. (a) Experimental setup. (b) Experimental procedure. The mice were subjected to 24‐h long recordings over 9 days. Red dots indicate sleep staging information and black dots the lack of sleep staging. Striped hours indicate periods where sleep deprivation was administered by a motorised wheel. *Source:* The figure was adapted from the article of Han et al. (2022).

The sample consists of nine mice (*Mus musculus, C57BL/6 and 129S4/SvJae hybrids*; Han et al. 2022), exposed to a 9‐day‐long continuous EcoG recording containing a sleep deprivation paradigm with baseline, sleep deprivation, and recovery phases. At the time of surgery, the mice were 12 weeks old. Consecutive to implanting the electrodes, 5 days of habituation, then 9 days (Figure 1) of recording was conducted. Each day consisted of an alternating light and dark cycle (with freely available food and water), namely, from 00:00 to 06:00 the lights were on, from 06:00 to 18:00 the lights were off and then on again from 18:00 to 24:00. The first day, serving as a baseline (BL), the mice were free roaming. Afterwards, for 5 days they were sleep deprived (sleep restriction days, SR) using a motorised wheel (14 cm in diameter, 5.8 cm in width, Lafayette Instrument, IN, USA) starting at 00:00 until 18:00, after which the mice were free roaming between 18:00 and 24:00. The last 3 days served as the recovery phase (recovery days, R), in which, following the same light and dark cycle, the mice were free roaming in their cages.

Recordings were conducted in a sound‐proof Faraday cage. The frontal and parietal registers were measured in a bipolar scheme referred to the left interparietal bone with the ground electrode on the right interparietal bone. The signals were digitised after amplification (analog amplifier: QP511, Grass Technologies, West Warwick, RI, USA) using an analog‐digital converter (Digidata 1440A, Molecular Devices, Sunnyvale, CA, USA). The sampling frequency was specified at 500 Hz, with a high‐pass filter at 0.3 Hz, a low‐pass filter at 100 Hz, and a 60 Hz hardware notch filter applied.

### 3.2 Ethics Statement

All procedures involving animal experimentation adhered strictly to ethical guidelines and were reviewed and approved by the Institutional Animal Care and Use Committee (IACUC) of the Korea Institute of Science and Technology (KIST). The approval was granted under the protocol identification number AP‐2014L7002, ensuring compliance with established standards for the humane treatment and care of research animals (Han et al. 2022).

### 3.3 Artefact Rejection and Sleep Staging

Artefact rejection was done manually, focusing on the harmonics of the network noise (60 Hz); on average, data loss was below 5%. The 24‐h long recording sessions were partitioned into 1‐h long segments. Out of the nine mice, subject 6 had faulty recordings on the frontal channel; therefore, sleep scoring is missing for this animal. Accordingly, subject 6 was omitted from further analysis.

Sleep staging was performed by manual scoring of conventional ECoG recordings (Han et al. 2022; Kim et al. 2020). Specifically, ECoG data from the frontal cortex were divided into 10‐s epochs and manually scored into three vigilance states (wake, NREM sleep, or REM sleep) using the SleepSign software. This scoring was informed by synchronized motion sensor data to help distinguish sleep states.

### 3.4 Power Spectrum

Twenty‐four hour recordings were divided into 1‐h long segments, then spectral analysis was conducted separately, for each hour's REM, NREM and WAKE episodes, without considering sleep staging information. In other words, each hour had four corresponding power spectra computed: NREM, REM, WAKE and finally an ALL segment. The latter included all non‐artefactual ECoG data regardless of sleep stage classification. This was introduced to examine signal dynamics in a stage‐independent manner. Power spectral density (PSD) was estimated using Welch's method with a Hann window (*n* = 2000 point symmetric window, 4 s), 50% overlap, and the number of discrete Fourier transform points used in the PSD estimate specified at 2000.

### 3.5 Scale‐Free Parameters of the Power Spectrum

The concept of scale‐invariance (scale‐free) is formalized such that

(1) $$\frac{f\left(\mathrm{kx}\right)}{f\left(x\right)}=g\left(k\right)$$

where *k* is a scaling factor, since the scaling does not depend on *x*. In practice this translates to “scaling the argument of the function is equivalent to a proportional scaling of the function itself” (Zimmern 2020). This holds for the function $f\left(x\right)={\mathrm{Cx}}^{-\alpha }$, where *α* is the scaling exponent, essentially describing a power law, which is a density function in the form of $P\left(x\right)={\mathrm{Cx}}^{-\alpha }$. Note that $\frac{f\left(\mathrm{kx}\right)}{f\left(x\right)}=\frac{{\mathrm{Ck}}^{-\alpha }x^{-\alpha }}{{\mathrm{Cx}}^{-\alpha }}=k^{-\alpha }$.

In the log–log space, an exponent is rendered a coefficient, transforming the function being investigated into a first‐degree polynomial. A method, called fitting oscillations and one over f (FOOOF), used to extract the power‐law exponent has been developed by Donoghue et al. (2020). It is a data‐driven method with the purpose of describing the power spectrum on the assumption of the brain expressing scale‐invariant, mono‐fractal properties. It is mathematically expressed as:

(2) $$P\left(f\right)=L\left(f\right)+\sum_{n}G_{n}\left(f\right)$$

where *P*(*f*) is the sum of possibly multiple Gaussians *G*_*n*_(*f*) and the aperiodic component *L*(*f*), which is parametrized as a Lorentzian function:

(3) $$L\left(f\right)=b-\log\left(k+f^{\alpha }\right)$$

where *b* is the spectral intercept, *α* is the spectral slope, and *k* is the spectral knee. FOOOF computes the following parameters: (1) the scaling exponent (spectral slope); (2) the whitened peak, (3) width and (4) the expected value of multiple spectral peaks; (5) spectral intercept and (6) the spectral knee. The spectral exponent (also called spectral slope) refers to the background neural activity, the spectral peaks are the oscillatory activities superimposed over the background, the spectral intercept is the offset of the fitted curve, and the spectral knee is the point at which the power spectrum takes on its characteristic‐coloured noise form on the logarithmically scaled space. The MATLAB implementation of the FOOOF fitting algorithm was applied over the frequency range of 0.5–48 Hz, with the following input parameters: width of peaks = default; maximum number of peaks = default; minimum height of peaks = default; peak threshold = default; aperiodic mode = ‘knee’. The present study focuses on the spectral slope, the spectral intercept, and the spectral knee. For the constructed models, *R* ^2^ values never dropped below 0.9.

### 3.6 Informational Entropy‐Based Characterisation of Neurophysiological Signals

Entropy denotes the regularity of a signal—a highly ordered and therefore predictable signal is said to have low entropy, while an unpredictable and complex signal is characterised by high values of entropy. A measure of entropy, called Irregularity Index (*II*) has been proposed to measure EEG complexity (Inouye et al. 1991). In practice, it involves calculating Shannon entropy (Shannon 1948) with log base 2 of the relative power of the time series signal, such that:

(4) $$\mathrm{II}=-\sum_{k=1}^{N}P_{k}\;\log_{2}\left(P_{k}\right)$$

where *k* corresponds to the frequency *λ*_*k*_ and *P*_*k*_ is given by:

(5) $$P_{k}=\frac{{\left|\lambda_{k}\right|}^{2}}{\sum {\left|\lambda_{i}\right|}^{2}}$$

|*λ*_*k*_|^2^ being the power spectrum of a time‐series at frequency *λ*_*k*_. However, this method lacks comparability—reflecting on this issue, namely that Shannon entropy does not yield a standardised measure, Zaccarelli et al. (2013) proposed and validated an index called Normalised Spectral Entropy (*H*_*Sn*_), normalised to the range of values between 0 and 1, such that:

(6) $$H_{\mathrm{Sn}}=\frac{\mathrm{II}}{\log_{2}\left(N\right)}$$

where *II* is the Irregularity Index (or spectral entropy) and *N* is the number of frequency bins. The normalisation ensures that the entropy value lies between 0 (representing a perfectly predictable or periodic signal) and 1 (representing a maximally random or flat spectrum). Important to note that in the framework of criticality, a maximally random system lacks structure, therefore should bear no information content and it should be assigned an entropy value of zero. We attempt to resolve the contradiction between this and our choice of *H*_*Sn*_ by our motive to uniformalise our analysis and keep it in the frequency domain and by previous empirical results. Namely, that Spectral entropy has been used in drowsiness detection (Sriraam et al. 2016), it has been associated with sleep deprivation in humans (Helakari et al. 2023). The present study utilises the EntropyHub MATLAB toolbox (Flood and Grimm 2021).

### 3.7 Data Analysis

Sleep scoring (WAKE, REM and NREM) was performed on Day 1 (baseline—BL), Day 2 (sleep restriction—SR1), Day 4 (sleep restriction—SR3), Day 6 (sleep restriction—SR5), Day 7 (recovery—R1), and Day 9 (recovery—R3). Due to missing data and insufficient NREM duration, the 18:00 to 19:00 time intervals were excluded from all subjects. Consequently, our analysis focused on the 19:00 to 24:00 periods of the specified days, incorporating associated sleep scoring information and analyzing NREM, REM and WAKE episodes separately. Additionally, a fourth analysis was conducted across all non‐artefactual ECoG segments on an hourly basis, without regard to sleep–wake stage classification.

The parietal EEG channel was used for analysis (the frontal channel was excluded due to redundancy). Repeated‐measures analyses were conducted using TIBCO Statistica (version 14.0.1.25). The primary statistical model was a repeated‐measures ANOVA with two within‐subject factors: DAY (six levels: BL, SR1, SR3, SR5, R1 and R3) and HOUR (five levels: 19–20, 20–21, 21–22, 22–23 and 23–24). The factor HOUR was nested within DAY, reflecting the hierarchical temporal structure of the experiment. The model assumed sphericity and normally distributed residuals, using the identity link function, as is standard in the GLM framework for repeated‐measures ANOVA.

Significant main effects were followed up with Bonferroni‐adjusted post hoc tests, and *α* < 0.01 was used as the threshold for statistical significance. The outcome metrics analysed were: (1) summed and natural log‐transformed spectral power within the SWA frequency band (0.75–4.5 Hz), (2) the spectral slope, (3) the spectral intercept, (4) the spectral knee, and (5) normalised spectral entropy. These parameters were selected to assess both traditional and broadband/fractal properties of the neurophysiological signal in relation to sleep homeostasis.

## 4 Results

### 4.1 Time Spent in Wakefulness, NREM and REM

**FIGURE 2:**
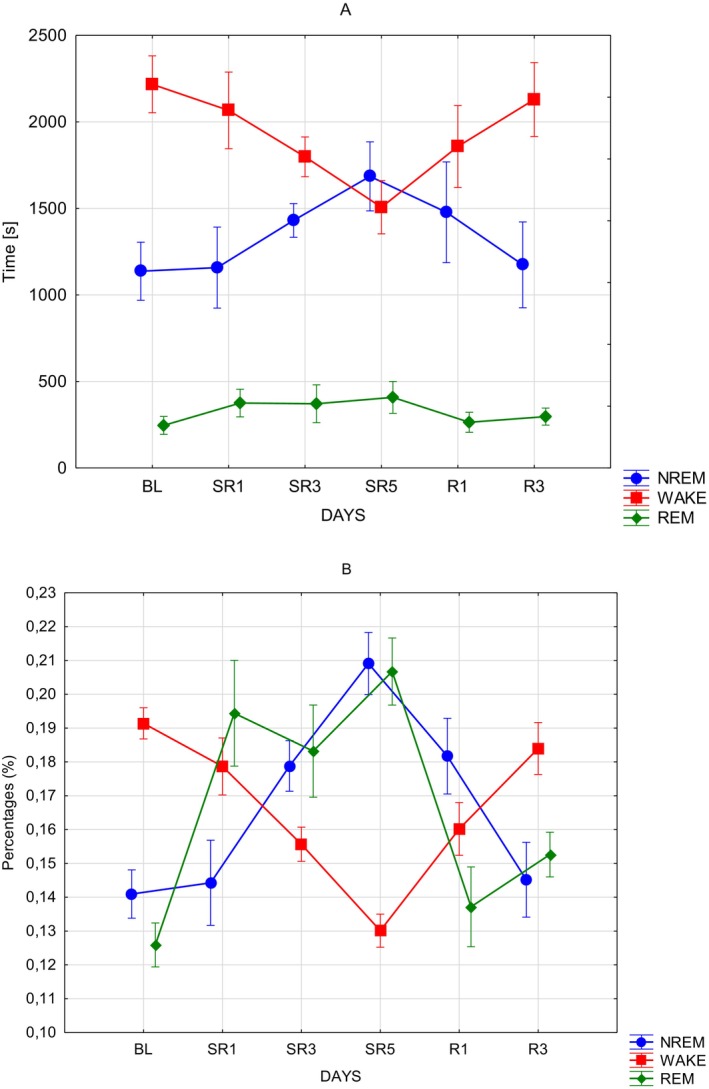
(A, B) Time spent in distinct behavioural states (WAKEFULNESS, NREM and REM) as functions of the experimental intervention, in (A) expressed in second and in (B) in percentages. Note the exclusive focus on hours 19:00–24:00 (see details in Section 3). Error bars denote standard errors. BL = baseline; *R* = recovery; SR = sleep restriction.

Table 1A–C shows the descriptive statistics (sample size, arithmetic mean, minimum, maximum and standard deviation) of time spent in WAKE, NREM and REM phases, respectively, during the sleep scored days in each hour from 19:00 to 24:00. Effective hypothesis decomposition revealed that NREM episodes varied significantly over the experimental days (DAYS: *F*(5, 35) = 6.0827; *p* = 0.0004; *η*_*p*_^2^ = 0.46). More precisely, comparing BL to SR5 (*t* = −4.2907; *p* = 0.002; *d* = −0.912), SR1 to SR5 (*t* = −4.1263; *p* = 0.003; *d* = −0.877), and SR5 to R3 (*t* = 4.0011; *p* = 0.004; *d* = 0.851) yielded significant results. In addition, statistically significant differences were observed in terms of time spent in wakefulness (WAKE) throughout the experimental days (Figure 2; DAYS: *F*(5, 35) = 9.8923; *p* < 0.00001; *η*_*p*_^2^ = 0.59). Bonferroni corrected post hoc tests of the DAYS independent variable revealed statistically significant differences contrasting BL sleep to SR3 (*t* = 3.561; *p* = 0.0163; *d* = 0.826) and SR5 (*t* = 6.0363; *p* < 0.001; *d* = 1.4), SR1 to SR5 (*t* = 4.7572; *p* < 0.001; *d* = 1.103), and SR5 to R3 (*t* = −5.2884; *p* < 0.001; *d* = −1.227). Considering REM phase, similarly, only the DAYS independent variable was significant (DAYS: *F*(5, 35) = 7.6155; *p* < 0.001; *η*_*p*_^2^ = 0.52). Post hoc test showed significant differences on the following comparisons: BL versus SR1 (*t* = −3.7864; *p* = 0.0086; *d* = −0.827), SR3 (*t* = −3.6622; *p* = 0.0123; *d* = −0.801) and SR5 (*t* = −4.7367; *p* < 0.001; *d* = −1.035); SR1 versus R1 (*t* = 3.2528; *p* = 0.038; *d* = 0.711); and SR5 versus R1 (*t* = 4.2031; *p* = 0.0026; *d* = 0.918) and R3 (*t* = 3.2455; *p* = 0.0387; *d* = 0.709).

**TABLE 1:** A descriptive statistics of time (min) spent in WAKE, NREM and REM.

Panel A: WAKE					
	18:00–19:00	19:00–20:00	20:00–21:00	21:00–22:00	22:00–23:00
Baseline	31.04 (9.99)	38.33 (6.53)	35.72 (7.23)	35.67 (5.52)	38.25 (7.97)
Sleep Restriction 1	19.12 (12.2)	36.58 (8.35)	33.81 (6.08)	31.9 (11.71)	37.77 (7.62)
Sleep Restriction 3	11.87 (11.4)	26.06 (9.99)	26.88 (9.33)	33.16 (6.47)	35.68 (4.02)
Sleep Restriction 5	8.13 (5.93)	20.58 (9.49)	22.75 (8.97)	25.70 (5.42)	25.75 (11.4)
Sleep Recovery 1	25.04 (14.5)	34.06 (9.07)	30.75 (11.2)	26.77 (9.37)	34.17 (10.3)
Sleep Recovery 3	29.14 (14.1)	37.02 (7.89)	34.38 (10.5)	32.39 (8.52)	38.75 (3.54)

Panel B: NREM					
	18:00–19:00	19:00–20:00	20:00–21:00	21:00–22:00	22:00–23:00
Baseline	25.83 (11.42)	18.46 (6.74)	19.83 (7.16)	20.19 (6.38)	17.17 (8.87)
Sleep Restriction 1	35.73 (14.83)	16.79 (9.26)	21.83 (6.91)	22.4 (13.51)	13.71 (9.97)
Sleep Restriction 3	45.69 (12.49)	27.81 (10.73)	28.06 (9.62)	20.54 (8.85)	16.33 (7.08)
Sleep Restriction 5	52.96 (7.75)	33.98 (11.96)	30.98 (12.96)	27.31 (8.22)	26.83 (13.82)
Sleep Recovery 1	32.27 (16.16)	21.79 (9.88)	25.04 (13.01)	29.4 (11.62)	20.58 (11.45)
Sleep Recovery 3	27.33 (15.64)	18.02 (8.97)	20.88 (12.9)	23.02 (10.14)	16.04 (3.62)

Panel C: REM					
	18:00–19:00	19:00–20:00	20:00–21:00	21:00–22:00	22:00–23:00
Baseline	3.13 (2)	3.21 (1.6)	4.44 (2.08)	4.15 (2.32)	4.58 (1.88)
Sleep Restriction 1	5.15 (3.37)	6.63 (3.02)	4.35 (1.66)	5.69 (2.74)	8.52 (3.26)
Sleep Restriction 3	2.44 (2.24)	6.13 (2.99)	5.06 (1.73)	6.29 (3.25)	7.98 (3.7)
Sleep Restriction 5	1.96 (2.04)	5.44 (3.09)	6.27 (4.19)	6.98 (4.17)	7.42 (2.97)
Sleep Recovery 1	2.69 (2.15)	4.15 (1.25)	4.21 (2.12)	3.83 (2.39)	5.25 (1.73)
Sleep Recovery 3	3.52 (2.14)	4.96 (1.61)	4.75 (2.94)	4.58 (2.1)	5.21 (1.5)

*Note:* Table 1A–C contains the descriptive statistics of time (min) spent in WAKE, NREM and REM in the following format: mean (standard deviation).

In other words, the mice appeared to spend more time in NREM and REM sleep as a response to sleep deprivation, which decrement returned to baseline values during recovery days. On the other hand, WAKE appeared to follow an inverse pattern; that is, it increased during sleep‐deprived days, then renormalized.

### 4.2 SWA

**FIGURE 3:**
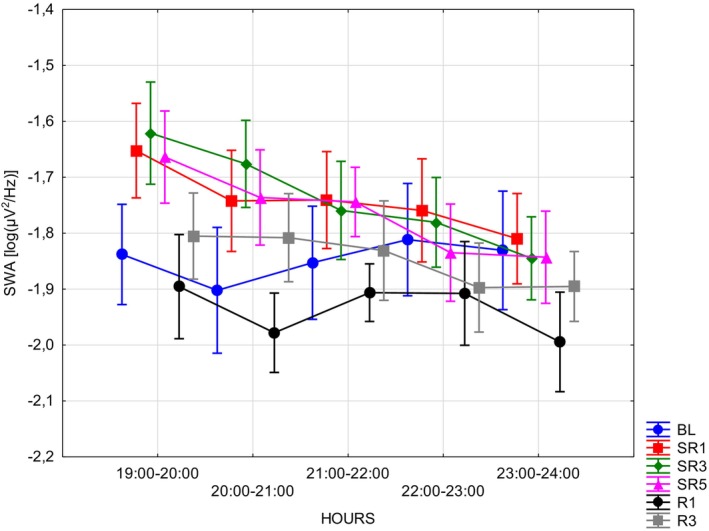
NREM sleep ECoG SWA as a function of the experimental intervention and time of day. Error bars denote standard errors. BL = baseline; *R* = recovery; SR = sleep restriction.

In terms of SWA no significant effects of sleep deprivation or recovery hour emerged for WAKE (Table 2A) or REM (Table 2C), whereas for NREM (Table 2B) scored segments, both independent variables (DAYS: *F*(5, 35) = 3.6227; *p* = 0.00957; *η*_*p*_^2^ = 0.34, HOURS: *F*(4, 28) = 10.04; *p* = 0.00004; *η*_*p*_^2^ = 0.58) and their interaction (*F*(20, 140) = 2.0156; *p* = 0.00983; *η*_*p*_^2^ = 0.22) were significant (Figure 3). Throughout the experimental days, the following comparisons were significant: SR1 versus R1 (mean diff = 0.1955; SE = 0.058; *p* = 0.0298) and SR3 versus R1 (mean diff = 0.2; SE = 0.058; *p* = 0.0241). Post hoc tests of the HOURS independent variable showed significant differences contrasting the 19–20 versus 22–23 (mean diff = 0.085; SE = 0.02; *p* = 0.0021) and 23–24 (mean diff = 0.062; SE = 0.02; *p* < 0.0001) intervals, and the 20–21 versus 23–24 (mean diff = 0.063; SE = 0.02; *p* = 0.043) and 21–22 versus 23–24 (mean diff = 0.064; SE = 0.02; *p* = 0.0362) intervals. Post hoc tests of the interaction term are reported in Appendix A (Table A1). However, when all the scores (NREM, REM, WAKE; Table 2D) were considered, only the DAYS independent variable was found to be statistically significant (*F*(5, 35) = 5.1116; *p* = 0.00127; *η*_*p*_^2^ = 0.42). Post hoc tests revealed significant differences between BL and R1 (mean diff = 0.175; SE = 0.053; *p* = 0.037), and SR1 versus R1 (mean diff = 0.179; SE = 0.05; *p* = 0.0299) and R3 (mean diff = 0.234; SE = 0.05; *p* = 0.0016). That is to say, ECoG power in the low frequency range is enhanced as a result of sleep deprivation, but sleep deprivation‐related effects are mainly present during the NREM phase of the first few hours of sleep, which progressively become normalized (Figure 3).

**TABLE 2:** Descriptive statistics of SWA in WAKE, NREM, REM and ALL recorded periods.

Panel A: WAKE					
	19:00–20:00	20:00–21:00	21:00–22:00	22:00–23:00	23:00–24:00
Baseline	−2.87 (0.22)	−2.83 (0.3)	−2.69 (0.38)	−2.7 (0.12)	−2.59 (0.44)
Sleep Restriction 1	−2.75 (0.2)	−2.76 (0.25)	−2.78 (0.2)	−2.73 (0.16)	−2.54 (0.55)
Sleep Restriction 3	−2.73 (0.25)	−2.72 (0.28)	−2.72 (0.24)	−2.69 (0.39)	−2.69 (0.25)
Sleep Restriction 5	−2.74 (0.25)	−2.8 (0.3)	−2.67 (0.39)	−2.81 (0.25)	−2.8 (0.28)
Sleep Recovery 1	−2.86 (0.24)	−2.85 (0.22)	−2.69 (0.62)	−2.81 (0.24)	−2.79 (0.3)
Sleep Recovery 3	−2.82 (0.28)	−2.91 (0.32)	−2.79 (0.27)	−2.77 (0.25)	−2.84 (0.34)

Panel B: NREM					
	19:00–20:00	20:00–21:00	21:00–22:00	22:00–23:00	23:00–24:00
Baseline	−1.84 (0.25)	−1.9 (0.32)	−1.85 (0.29)	−1.81 (0.28)	−1.83 (0.3)
Sleep Restriction 1	−1.65 (0.24)	−1.74 (0.26)	−1.74 (0.25)	−1.76 (0.26)	−1.81 (0.23)
Sleep Restriction 3	−1.62 (0.26)	−1.68 (0.22)	−1.76 (0.25)	−1.78 (0.23)	−1.84 (0.21)
Sleep Restriction 5	−1.66 (0.23)	−1.74 (0.24)	−1.74 (0.18)	−1.83 (0.25)	−1.84 (0.23)
Sleep Recovery 1	−1.9 (0.26)	−1.98 (0.2)	−1.91 (0.15)	−1.91 (0.26)	−1.99 (0.25)
Sleep Recovery 3	−1.81 (0.22)	−1.81 (0.22)	−1.83 (0.25)	−1.9 (0.22)	−1.9 (0.18)

Panel C: REM					
	19:00–20:00	20:00–21:00	21:00–22:00	22:00–23:00	23:00–24:00
Baseline	−2.74 (0.26)	−2.79 (0.34)	−2.8 (0.24)	−2.71 (0.2)	−2.7 (0.24)
Sleep Restriction 1	−2.64 (0.3)	−2.73 (0.27)	−2.56 (0.32)	−2.74 (0.22)	−2.71 (0.27)
Sleep Restriction 3	−2.69 (0.28)	−2.73 (0.29)	−2.86 (0.27)	−2.77 (0.25)	−2.94 (0.21)
Sleep Restriction 5	−2.73 (0.24)	−2.75 (0.26)	−2.74 (0.16)	−2.77 (0.2)	−2.78 (0.23)
Sleep Recovery 1	−2.75 (0.27)	−2.88 (0.14)	−2.82 (0.2)	−2.93 (0.19)	−2.84 (0.13)
Sleep Recovery 3	−2.75 (0.24)	−2.77 (0.16)	−2.77 (0.17)	−2.82 (0.21)	−2.73 (0.27)

Panel D: ALL recorded periods					
	19:00–20:00	20:00–21:00	21:00–22:00	22:00–23:00	23:00–24:00
Baseline	−2.09 (0.24)	−2.18 (0.29)	−2.12 (0.24)	−2.06 (0.18)	−2.06 (0.29)
Sleep Restriction 1	−1.95 (0.23)	−2.06 (0.19)	−2.11 (0.28)	−2.03 (0.19)	−2.06 (0.26)
Sleep Restriction 3	−2.06 (0.18)	−2.12 (0.23)	−2.08 (0.26)	−2.06 (0.21)	−2.22 (0.16)
Sleep Restriction 5	−2.24 (0.22)	−2.25 (0.36)	−2.18 (0.17)	−2.24 (0.24)	−2.2 (0.25)
Sleep Recovery 1	−2.2 (0.27)	−2.33 (0.21)	−2.28 (0.33)	−2.23 (0.2)	−2.35 (0.31)
Sleep Recovery 3	−2.07 (0.18)	−2.17 (0.29)	−2.17 (0.21)	−2.13 (0.18)	−2.19 (0.23)

*Note:* Table 2A–D contains the descriptive statistics of SWA in WAKE, NREM, REM episodes, and when all scores were considered (ALL), in the following format: mean (standard deviation).

### 4.3 Spectral Intercept

**FIGURE 4:**
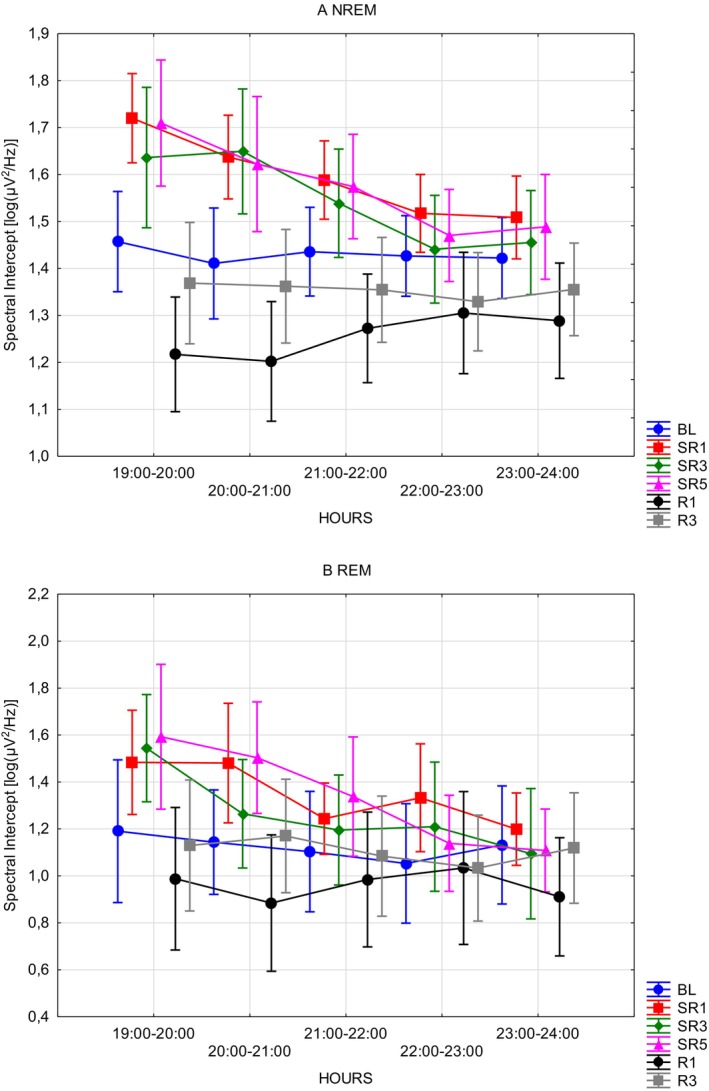
Sleep ECoG spectral intercepts as functions of the experimental intervention and time of day. (A) NREM sleep ECoG spectral intercepts. (B) REM sleep ECoG spectral intercepts. Error bars denote standard errors. BL = baseline; *R* = recovery; SR = sleep restriction.

In terms of the spectral intercept during WAKE (Table 3) periods only the DAYS independent variables were significant (*F*(5, 35) = 3.715; *p* = 0.0084; *η*_*p*_^2^ = 0.34), which according to the Bonferroni corrected post hoc test, indicated the difference between SR1 and R1 (mean diff = 0.24; SE = 0.06; *p* = 0.0055). However, for REM (Table 3B and Figure 4B) multiple significant main effects were found (DAYS: *F*(5, 20) = 6.3665; *p* = 0.0011; *η*_*p*_^2^ = 0.61, HOURS: *F*(4, 16) = 18.6054; *p* < 0.001; *η*_*p*_^2^ = 0.82). Post hoc tests showed significant comparisons for SR1 versus R1 (mean diff = 0.085; SE = 0.086; *p* = 0.002), SR3 versus R1 (mean diff = 0.301; SE = 0.0856; *p* = 0.031) and SR5 versus R1 (mean diff = 0.376; SE = 0.085; *p* = 0.0039); and for 19–20 versus 21–22 (mean diff = 0.163; SE = 0.0299; *p* < 0.001), 22–23 (mean diff = 0.18; SE = 0.0299; *p* < 0.001) and 23–24 (mean diff = 0.227; SE = 0.02; *p* < 0.001), and 20–21 versus 22–23 (mean diff = 0.011; SE = 0.02; *p* = 0.024) and 23–24 (mean diff = 0.147; SE = 0.0299; *p* = 0.0015). In addition, for NREM (Table 3B and Figure 4A), both independent variables (DAYS: *F*(5, 35) = 8.6869; *p* < 0.001; *η*_*p*_^2^ = 0.55, HOURS: *F*(4, 28) = 5.8955; *p* = 0.0014; *η*_*p*_^2^ = 0.45) and their interaction were found to be significant (Figure 4; *F*(20, 140) = 2.8006; *p* < 0.001; *η*_*p*_^2^ = 0.29); post hoc tests are reported in Appendix A (Table A2). Post hoc tests revealed significant differences between SR1 and R1 (mean diff = 0.33; SE = 0.06; *p* < 0.001) and R3 (mean diff = 0.24; SE = 0.64; *p* = 0.0013), between SR3 and R1 (mean diff = 0.28; SE = 0.06; *p* < 0.001), and finally between SR5 and R1 (mean diff = 0.32; SE = 0.06; *p* < 0.001). Lastly, for the aggregated segments (Table 3D; WAKE, NREM and REM included), significant differences were only observed throughout the experimental days (DAYS: *F*(5, 35) = 8.812; *p* < 0.001; *η*_*p*_^2^ = 0.54). More precisely, BL versus R1 (mean diff = 0.297; SE = 0.075; *p* = 0.005), SR1 versus SR5 (mean diff = 0.277; SE = 0.0757; *p* = 0.0101).

**TABLE 3:** Descriptive statistics of spectral intercept in WAKE, NREM, REM and ALL recorded periods.

Panel A: WAKE					
	19:00–20:00	20:00–21:00	21:00–22:00	22:00–23:00	23:00–24:00
Baseline	−0.32 (0.8)	−0.27 (0.71)	−0.17 (0.78)	−0.17 (0.65)	−0.23 (0.66)
Sleep Restriction 1	−0.02 (0.61)	−0.11 (0.67)	−0.14 (0.68)	0.14 (0.6)	−0.23 (0.45)
Sleep Restriction 3	−0.16 (0.65)	−0.15 (0.71)	−0.12 (0.68)	−0.09 (0.7)	−0.29 (0.58)
Sleep Restriction 5	−0.2 (0.73)	−0.17 (0.65)	−0.14 (0.69)	−0.18 (0.64)	0.01 (0.64)
Sleep Recovery 1	−0.37 (0.74)	−0.35 (0.76)	−0.41 (0.61)	−0.19 (0.64)	−0.24 (0.71)
Sleep Recovery 3	−0.13 (0.69)	−0.33 (0.66)	−0.19 (0.56)	−0.22 (0.69)	−0.19 (0.71)

Panel B: NREM					
	19:00–20:00	20:00–21:00	21:00–22:00	22:00–23:00	23:00–24:00
Baseline	1.46 (0.3)	1.41 (0.33)	1.44 (0.27)	1.43 (0.24)	1.42 (0.24)
Sleep Restriction 1	1.72 (0.27)	1.64 (0.25)	1.59 (0.24)	1.52 (0.23)	1.51 (0.25)
Sleep Restriction 3	1.64 (0.42)	1.65 (0.38)	1.54 (0.33)	1.44 (0.32)	1.46 (0.31)
Sleep Restriction 5	1.71 (0.38)	1.62 (0.41)	1.57 (0.31)	1.47 (0.28)	1.49 (0.32)
Sleep Recovery 1	1.22 (0.35)	1.2 (0.36)	1.27 (0.33)	1.31 (0.37)	1.29 (0.35)
Sleep Recovery 3	1.37 (0.37)	1.36 (0.34)	1.35 (0.32)	1.33 (0.3)	1.36 (0.28)

Panel C: REM					
	19:00–20:00	20:00–21:00	21:00–22:00	22:00–23:00	23:00–24:00
Baseline	0.9 (0.67)	0.91 (0.49)	0.86 (0.56)	0.85 (0.52)	0.94 (0.5)
Sleep Restriction 1	1.13 (0.62)	1.21 (0.57)	1.01 (0.42)	1.1 (0.51)	0.98 (0.4)
Sleep Restriction 3	1.23 (0.58)	1.04 (0.51)	1.06 (0.49)	1.01 (0.56)	0.96 (0.56)
Sleep Restriction 5	1.26 (0.69)	1.23 (0.64)	1.1 (0.57)	0.95 (0.46)	0.9 (0.43)
Sleep Recovery 1	0.69 (0.66)	0.63 (0.61)	0.84 (0.58)	0.77 (0.66)	0.67 (0.55)
Sleep Recovery 3	0.85 (0.61)	0.97 (0.56)	0.9 (0.5)	0.87 (0.44)	0.86 (0.55)

Panel D: ALL recorded periods					
	19:00–20:00	20:00–21:00	21:00–22:00	22:00–23:00	23:00–24:00
Baseline	0.9 (0.52)	0.84 (0.42)	0.89 (0.44)	0.97 (0.26)	0.87 (0.29)
Sleep Restriction 1	1.22 (0.33)	1.02 (0.38)	0.94 (0.45)	1.13 (0.35)	0.82 (0.35)
Sleep Restriction 3	0.84 (0.57)	0.82 (0.41)	0.93 (0.54)	0.96 (0.51)	0.67 (0.47)
Sleep Restriction 5	0.65 (0.77)	0.72 (0.75)	0.77 (0.54)	0.79 (0.44)	0.88 (0.42)
Sleep Recovery 1	0.6 (0.6)	0.48 (0.58)	0.41 (0.54)	0.74 (0.43)	0.55 (0.56)
Sleep Recovery 3	0.87 (0.56)	0.72 (0.61)	0.72 (0.37)	0.87 (0.42)	0.82 (0.41)

*Note:* Table 3A–D contains the descriptive statistics of Spectral Intercept in WAKE, NREM, REM episodes, and when all scores were considered (ALL), in the following format: mean (standard deviation).

### 4.4 Spectral Slope

**FIGURE 5:**
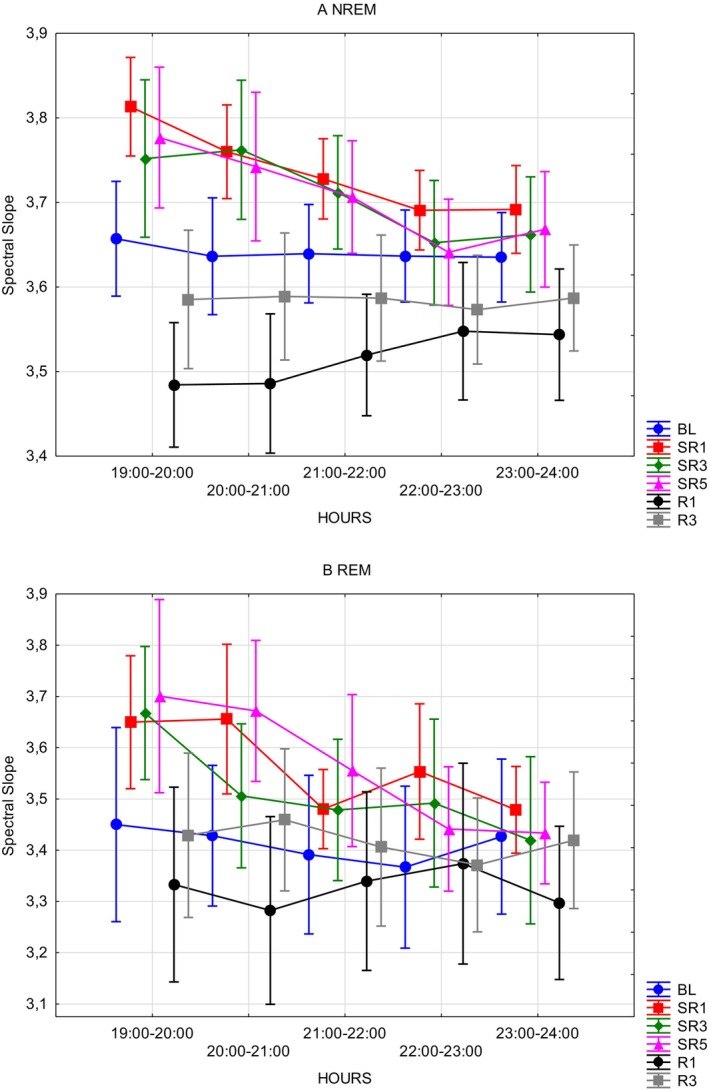
Sleep ECoG spectral slopes as functions of the experimental intervention and times of day. (A) NREM sleep ECoG spectral slopes. (B) REM sleep ECoG spectral slopes. Error bars denote standard errors. The ordinate axis represents the absolute value of the spectral slope. BL = baseline; *R* = recovery; SR = sleep restriction.

The spectral slopes of the ECoG records measured during the WAKE (Table 4A) segments did not show significant sleep deprivation‐related effects. On the other hand, during REM (Table 4C and Figure 5B) segments, both main effects (DAYS: *F*(5, 20) = 6.4481; *p* = 0.001; *η*_*p*_^2^ = 0.62; HOURS: *F*(4, 16) = 14.6874; *p* = 0.00003; *η*_*p*_^2^ = 0.78) were significant; however, their interaction (*F*(20, 80) = 1.9803; *p* = 0.01714; *η*_*p*_^2^ = 0.34) was only marginallysignificant. Post hoc tests are reported in Appendix A (Table A3). More precisely, the comparisons of SR1 versus R1 (mean diff = 0.238; SE = 0.05; *p* = 0.003), SR3 versus R1 (mean diff = 0.187; SE = 0.053; *p* = 0.032), and SR5 versus R1 (mean diff = 0.235; SE = 0.05; *p* = 0.0039) were found to be significant. Post hoc tests of the HOURS independent variable also revealed significant differences: 19–20 versus 21–22 (mean diff = 0.096; SE = 0.0193; *p* = 0.0013), 22–23 (mean diff = 0.105; SE = 0.0193; *p* = 0.0005), 23–24 (mean diff = 0.125; SE = 0.0193; *p* < 0.0001), and 20–21 versus 23–24 (mean diff = 0.088; SE = 0.019; *p* = 0.0031). An analysis with identical parameters was repeated for the NREM (Table 4B and Figure 5A) segments, which revealed significant main effects of DAYS (*F*(5, 35) = 10.905; *p* < 0.00001; *η*_*p*_^2^ = 0.61), HOURS (*F*(4, 28) = 4.214; *p* = 0.00854; *η*_*p*_^2^ = 0.37) and their interaction (*F*(20, 140) = 2.624; *p* = 0.00053; *η*_*p*_^2^ = 0.27) was only marginally significant; post hoc tests are reported in Appendix A (Table A4). Post hoc tests revealed significant differences between BL and R1 (mean diff = 0.12; SE = 0.36; *p* = 0.0247), SR1 and R1 (mean diff = 0.22; SE = 0.037; *p* ≤ 0.001), R3 (mean diff = 0.15; SE = 0.03; *p* = 0.002), SR3 and R1 (mean diff = 0.19; SE = 0.036; *p* < 0.001), R3 (mean diff = 0.12; SE = 0.036; *p* < 0.027), and lastly, between SR5 and R1 (mean diff = 0.19; SE = 0.036; *p* < 0.001), R3 (mean diff = 0.12; SE = 0.036; *p* = 0.028). Furthermore, post hoc testing of the HOURS independent variable revealed the 19–20 versus 22–23 (mean diff = 0.054; SE = 0.015; *p* = 0.014) comparison to be marginally significant. Last, but not least, the aggregated segments (Table 4D; unifying WAKE, NREM and REM) did indicate significant differences only for the DAYS independent variable (*F*(5, 35) = 9.385; *p* < 0.001; *η*_*p*_^2^ = 0.5727). Significant post hoc comparisons are the following: BL versus SR1 (mean diff = 0.236; SE = 0.05; *p* = 0.0005), SR1 versus SR5 (mean diff = 0.19; SE = 0.05; *p* < 0.0001) and R1 (mean diff = 0.3227; SE = 0.05; *p* = 0.005), SR3 versus R1 (mean diff = −0.1683; SE = 0.05; *p* = 0.02). In other words, the power spectrum appeared to respond with enhanced slow to high frequency ratios as captured by the steeper fitted curves to the ECoG spectra of sleep periods following sleep deprivation (SR1) relative to baseline sleep (BL).

**TABLE 4:** Descriptive statistics of spectral slope in WAKE, NREM, REM and ALL recorded periods.

Panel A: WAKE					
	19:00–20:00	20:00–21:00	21:00–22:00	22:00–23:00	23:00–24:00
Baseline	2.43 (0.55)	2.48 (0.47)	2.53 (0.54)	2.54 (0.44)	2.49 (0.43)
Sleep Restriction 1	2.66 (0.4)	2.58 (0.44)	2.55 (0.44)	2.77 (0.43)	2.52 (0.29)
Sleep Restriction 3	2.54 (0.41)	2.56 (0.45)	2.58 (0.42)	2.61 (0.46)	2.48 (0.36)
Sleep Restriction 5	2.5 (0.48)	2.54 (0.42)	2.56 (0.44)	2.53 (0.4)	2.69 (0.45)
Sleep Recovery 1	2.4 (0.48)	2.42 (0.5)	2.33 (0.38)	2.51 (0.41)	2.49 (0.46)
Sleep Recovery 3	2.56 (0.45)	2.42 (0.43)	2.5 (0.35)	2.49 (0.46)	2.52 (0.46)

Panel B: NREM					
	19:00–20:00	20:00–21:00	21:00–22:00	22:00–23:00	23:00–24:00
Baseline	3.66 (0.19)	3.64 (0.2)	3.64 (0.16)	3.64 (0.15)	3.64 (0.15)
Sleep Restriction 1	3.81 (0.16)	3.76 (0.16)	3.73 (0.13)	3.69 (0.13)	3.69 (0.15)
Sleep Restriction 3	3.75 (0.26)	3.76 (0.23)	3.71 (0.19)	3.65 (0.21)	3.66 (0.19)
Sleep Restriction 5	3.78 (0.24)	3.74 (0.25)	3.71 (0.19)	3.64 (0.18)	3.67 (0.19)
Sleep Recovery 1	3.48 (0.21)	3.49 (0.23)	3.52 (0.2)	3.55 (0.23)	3.54 (0.22)
Sleep Recovery 3	3.59 (0.23)	3.59 (0.21)	3.59 (0.21)	3.57 (0.18)	3.59 (0.18)

Panel C: REM					
	19:00–20:00	20:00–21:00	21:00–22:00	22:00–23:00	23:00–24:00
Baseline	3.27 (0.42)	3.28 (0.31)	3.23 (0.36)	3.23 (0.33)	3.31 (0.31)
Sleep Restriction 1	3.43 (0.38)	3.48 (0.34)	3.34 (0.24)	3.41 (0.3)	3.33 (0.25)
Sleep Restriction 3	3.47 (0.35)	3.36 (0.31)	3.38 (0.3)	3.36 (0.35)	3.33 (0.33)
Sleep Restriction 5	3.48 (0.44)	3.49 (0.4)	3.4 (0.35)	3.31 (0.28)	3.29 (0.26)
Sleep Recovery 1	3.14 (0.42)	3.11 (0.39)	3.24 (0.36)	3.19 (0.41)	3.14 (0.34)
Sleep Recovery 3	3.24 (0.38)	3.32 (0.34)	3.28 (0.32)	3.26 (0.27)	3.25 (0.33)

Panel D: ALL recorded periods					
	19:00–20:00	20:00–21:00	21:00–22:00	22:00–23:00	23:00–24:00
Baseline	3.26 (0.36)	3.24 (0.28)	3.25 (0.3)	3.32 (0.18)	3.24 (0.18)
Sleep Restriction 1	3.48 (0.23)	3.34 (0.25)	3.29 (0.29)	3.42 (0.23)	3.22 (0.23)
Sleep Restriction 3	3.2 (0.39)	3.2 (0.25)	3.29 (0.34)	3.32 (0.34)	3.12 (0.29)
Sleep Restriction 5	3.07 (0.52)	3.13 (0.49)	3.17 (0.35)	3.17 (0.29)	3.26 (0.29)
Sleep Recovery 1	3.06 (0.4)	2.99 (0.39)	2.91 (0.36)	3.15 (0.28)	3.03 (0.38)
Sleep Recovery 3	3.24 (0.39)	3.14 (0.4)	3.14 (0.24)	3.25 (0.28)	3.21 (0.27)

*Note:* Table 4A–D contains the descriptive statistics of Spectral Slope in WAKE, NREM, REM episodes, and when all scores were considered (ALL), in the following format: mean (standard deviation).

**FIGURE 6:**
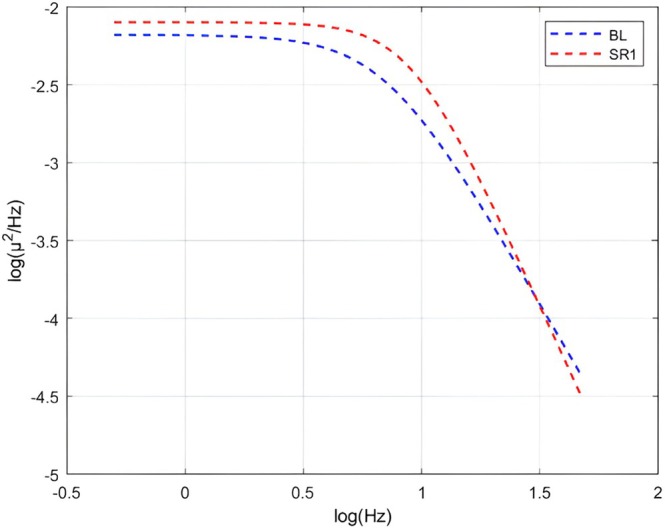
Aperiodic fits of subject 1 over 19:00–20:00 BL and SR1 recordings. Figure 6 depicts the aperiodic fits of subject one during the second hour (19:00–20:00) of the baseline sleep (BL) and the first sleep following sleep deprivation (SR1). The aperiodic fit refers to the model fitted over the power spectrum which omits the spectral peaks (oscillatory activity) and only contains the neural background activity (also called spectral exponent or spectral slope). The figure showcases the difference between two power spectra, one of which being steeper (sleeping after sleep deprivation in this case).

### 4.5 Spectral Knee

**FIGURE 7:**
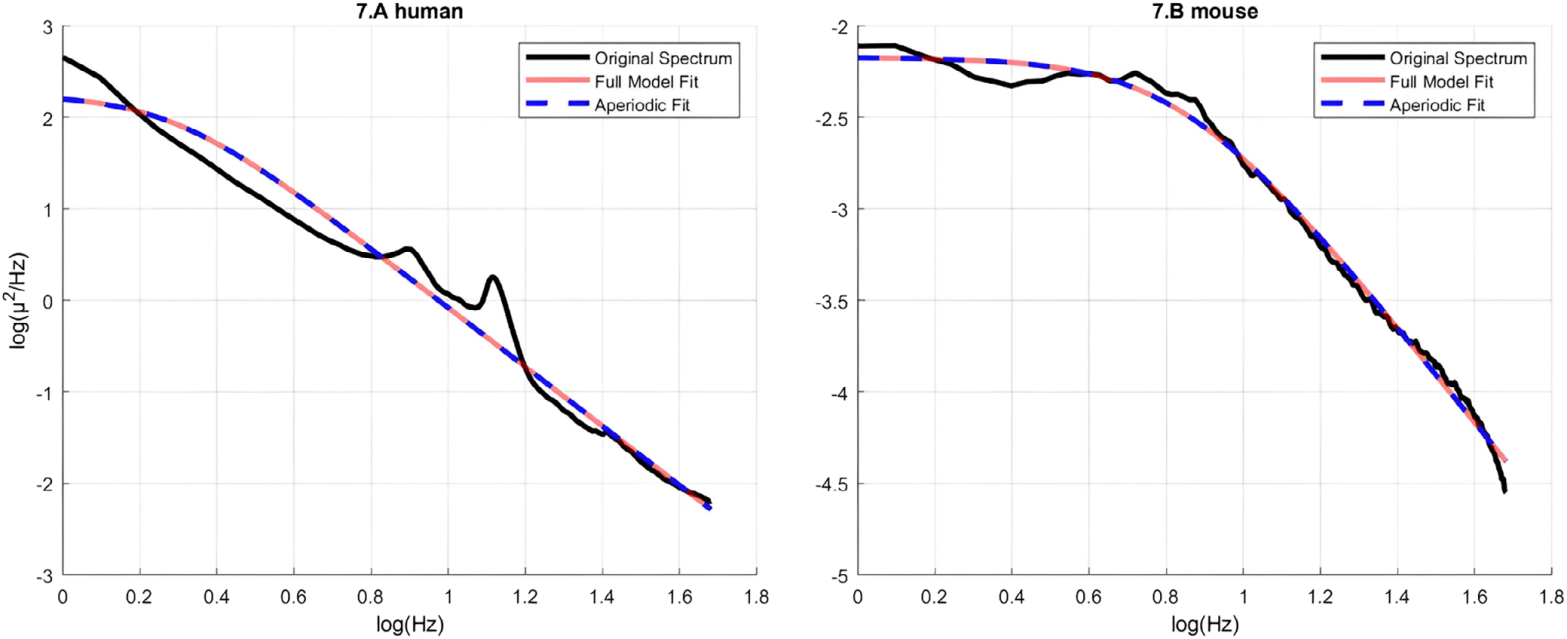
The depiction of the comparative and species‐specific perspective of the spectral knee issue in surface electrophysiological signals. (A) The log–log representation of the scalp‐recorded EEG of a healthy human volunteer (age: 27 years, gender: female, recording location: C4‐A1A2) and its parametrization performed by the FOOOF procedure. Note the lack of the spectral knee in the displayed frequency range (1–48 Hz). (B) The log–log representation of the ECoG of a mouse (subject 1, Baseline sleep during 19:00–20:00 h, recording location: CP6—interparietal bone) and its' parametrization performed by the FOOOF procedure. Note the prominent spectral knee around 9 Hz frequency.

Table 5A–C details the descriptive of the spectral knee, that is, the point at which the log–log power spectrum linearizes. It appears that the spectral knee is shifted to the right as opposed to human data (Figure 7A,B). More precisely, it averages around 9 Hz with a range of 7–11 Hz in mice. As a result of the substantial right shift, the spectral slope is unfit to describe slow oscillations.

**TABLE 5:** Descriptive statistics of Spectral Knee in WAKE, NREM, REM, and ALL recorded periods.

Panel A: WAKE					
	19:00–20:00	20:00–21:00	21:00–22:00	22:00–23:00	23:00–24:00
Baseline	6.8 (2.12)	6.77 (2.11)	6.65 (1.58)	6.81 (1.63)	6.39 (1.18)
Sleep Restriction 1	7.44 (1.57)	7.21 (2.11)	7.19 (1.93)	7.85 (1.53)	6.42 (1.25)
Sleep Restriction 3	6.98 (1.98)	6.96 (2.32)	6.98 (1.62)	6.99 (2.14)	6.49 (1.41)
Sleep Restriction 5	6.89 (2.04)	7.09 (2.05)	6.86 (1.69)	7.22 (2.05)	7.55 (2.09)
Sleep Recovery 1	6.59 (2.44)	6.63 (2.47)	6.28 (2.21)	7.11 (2.01)	6.85 (2.25)
Sleep Recovery 3	7.29 (2.2)	7.1 (1.94)	7.09 (1.51)	6.96 (1.78)	7.18 (1.78)

Panel B: NREM					
	19:00–20:00	20:00–21:00	21:00–22:00	22:00–23:00	23:00–24:00
Baseline	9.01 (0.61)	9 (0.7)	9.01 (0.45)	8.94 (0.39)	9 (0.39)
Sleep Restriction 1	9.49 (0.63)	9.38 (0.55)	9.31 (0.49)	9.16 (0.66)	9.22 (0.68)
Sleep Restriction 3	9.16 (0.78)	9.33 (0.87)	9.19 (0.91)	8.98 (1.05)	9.17 (1)
Sleep Restriction 5	9.45 (0.74)	9.35 (0.9)	9.33 (0.76)	9.39 (0.86)	9.39 (0.74)
Sleep Recovery 1	8.75 (1.02)	8.8 (1.02)	8.84 (0.77)	8.95 (0.85)	9.09 (0.89)
Sleep Recovery 3	8.88 (0.82)	8.79 (0.9)	8.86 (0.78)	8.93 (0.71)	8.97 (0.62)

Panel C: REM					
	19:00–20:00	20:00–21:00	21:00–22:00	22:00–23:00	23:00–24:00
Baseline	9.9 (1.34)	10.1 (1.1)	10.17 (1.19)	9.79 (1.11)	9.94 (0.94)
Sleep Restriction 1	10.2 (1.75)	10.65 (1.53)	9.98 (0.64)	10.41 (1.43)	10.07 (1.19)
Sleep Restriction 3	10.86 (1.74)	10.39 (1.43)	10.62 (1.27)	10.37 (1.44)	10.52 (1.33)
Sleep Restriction 5	11.07 (1.52)	10.9 (1.5)	10.61 (1.34)	10.2 (1.15)	10.04 (0.95)
Sleep Recovery 1	9.46 (1.78)	9.58 (1.3)	9.98 (1.39)	10.03 (1.47)	9.59 (1.19)
Sleep Recovery 3	9.93 (1.35)	10.19 (1.02)	10.08 (1.11)	10.13 (1.12)	9.81 (1.47)

Panel D: ALL recorded periods					
	19:00–20:00	20:00–21:00	21:00–22:00	22:00–23:00	23:00–24:00
Baseline	8.33 (0.97)	8.29 (0.87)	8.32 (0.64)	8.45 (0.53)	8.24 (0.5)
Sleep Restriction 1	8.99 (0.78)	8.66 (0.92)	8.59 (0.86)	8.87 (0.87)	8.15 (0.62)
Sleep Restriction 3	8.23 (1.37)	8.36 (1.25)	8.48 (1.12)	8.43 (1.3)	8.07 (1.08)
Sleep Restriction 5	8.14 (1.61)	8.32 (1.61)	8.31 (1.19)	8.64 (1.23)	8.69 (1.04)
Sleep Recovery 1	7.91 (1.44)	7.81 (1.39)	7.64 (1.3)	8.33 (1.14)	8.11 (1.3)
Sleep Recovery 3	8.25 (1.26)	8.12 (1.3)	8.15 (0.88)	8.4 (0.94)	8.42 (0.88)

*Note:* Table 5A–D contain the descriptive statistics of Spectral Knee in WAKE, NREM, REM episodes, and when all scores were considered (ALL), in the following format: mean (standard deviation).

**FIGURE 8:**
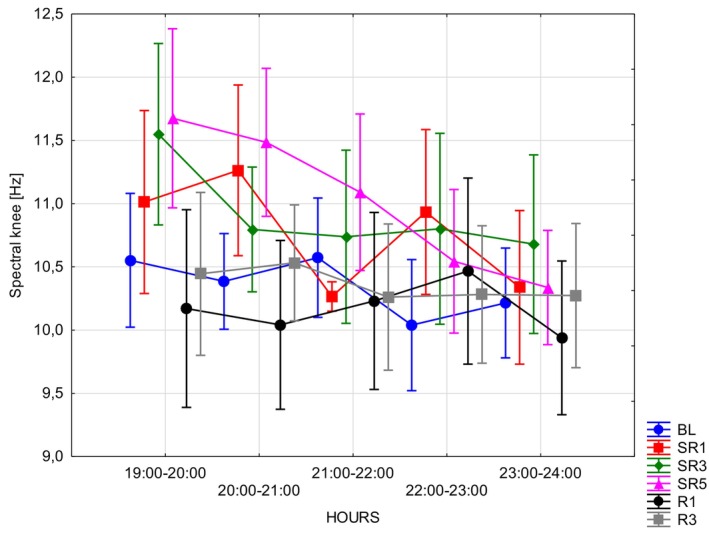
Spectral knees according to the FOOOF procedure as measured in ECoG recordings performed during REM periods of sleep. Error bars denote standard errors. BL = baseline, *R* = recovery; SR = sleep restriction.

The spectral knee for WAKE (Table 5A) and aggregated segments (Table 5D; unifying WAKE, NREM and REM) did not display significant main effects. However, hypothesis‐testing of REM segments (Table 5C and Figure 8) revealed a significant main effect of HOURS (*F*(4, 16) = 11.7127; *p* < 0.001; *η*_*p*_^2^ = 0.75), which was further dissected by post hoc tests showing that in terms of the levels of the HOURS repeated measure factor the following contrasts were significant or marginally significant: 19–20 versus 21–22 (mean diff = 0.3744; SE = 0.096; *p* = 0.0134), 22–23 (mean diff = 0.3897; SE = 0.097; *p* = 0.0096), 23–24 (mean diff = 0.6042; SE = 0.0966; *p* = 0.00011), and 20–21 versus 23–24 (mean diff = 0.454; SE = 0.09; *p* = 0.0023). However, the interaction between the independent variables was not found to be significant. Lastly, for NREM the spectral knee displayed no significant main effects.

### 4.6 Normalised Spectral Entropy

No significant nor marginally significant DAY and HOUR effects in terms of spectral entropy were revealed for WAKE, NREM, REM, or aggregated segments. Descriptive statistics of normalised spectral entropy are reported in Table 6A–D.

**TABLE 6:** Descriptive statistics of normalised spectral entropy in WAKE, NREM, REM and ALL recorded periods.

Panel A: WAKE					
	19:00–20:00	20:00–21:00	21:00–22:00	22:00–23:00	23:00–24:00
Baseline	0.648 (0.01)	0.6403 (0.02)	0.6014 (0.07)	0.6386 (0.01)	0.6296 (0.03)
Sleep Restriction 1	0.6405 (0.01)	0.641 (0.02)	0.6398 (0.01)	0.6398 (0.01)	0.626 (0.03)
Sleep Restriction 3	0.6406 (0.01)	0.6377 (0.02)	0.6384 (0.01)	0.6063 (0.07)	0.6366 (0.01)
Sleep Restriction 5	0.6436 (0.01)	0.643 (0.01)	0.6321 (0.03)	0.6452 (0.01)	0.6395 (0.01)
Sleep Recovery 1	0.6464 (0.01)	0.6451 (0.01)	0.623 (0.04)	0.6463 (0.01)	0.6428 (0.01)
Sleep Recovery 3	0.6438 (0.01)	0.6532 (0.01)	0.6338 (0.03)	0.6471 (0.01)	0.6462 (0.02)

Panel B: NREM					
	19:00–20:00	20:00–21:00	21:00–22:00	22:00–23:00	23:00–24:00
Baseline	0.6115 (0.01)	0.6123 (0.01)	0.6125 (0.01)	0.6111 (0.01)	0.6118 (0.01)
Sleep Restriction 1	0.6096 (0.01)	0.6106 (0.01)	0.6106 (0.01)	0.6097 (0.01)	0.6103 (0.01)
Sleep Restriction 3	0.605 (0)	0.6083 (0.01)	0.6081 (0.01)	0.6072 (0.01)	0.6104 (0.01)
Sleep Restriction 5	0.6107 (0.01)	0.6099 (0.01)	0.612 (0.01)	0.6132 (0.01)	0.6134 (0.01)
Sleep Recovery 1	0.6131 (0.01)	0.6146 (0.01)	0.6146 (0.01)	0.6136 (0.01)	0.6163 (0.01)
Sleep Recovery 3	0.6105 (0.01)	0.609 (0.01)	0.6102 (0.01)	0.6117 (0.01)	0.6127 (0.01)

Panel C: REM					
	19:00–20:00	20:00–21:00	21:00–22:00	22:00–23:00	23:00–24:00
Baseline	0.6364 (0.01)	0.6391 (0.01)	0.6426 (0.01)	0.6352 (0.01)	0.6358 (0.01)
Sleep Restriction 1	0.6329 (0.01)	0.636 (0.01)	0.612 (0.05)	0.6367 (0.01)	0.634 (0.01)
Sleep Restriction 3	0.6385 (0.02)	0.6376 (0.02)	0.6346 (0.01)	0.6394 (0.01)	0.6403 (0.01)
Sleep Restriction 5	0.6431 (0.01)	0.6375 (0.01)	0.6403 (0.01)	0.638 (0.01)	0.6375 (0.01)
Sleep Recovery 1	0.6363 (0.01)	0.6388 (0.01)	0.6375 (0.01)	0.6398 (0.01)	0.6365 (0.01)
Sleep Recovery 3	0.6369 (0.01)	0.6365 (0.01)	0.636 (0.01)	0.6379 (0.01)	0.6343 (0.01)

Panel D: ALL recorded periods					
	19:00–20:00	20:00–21:00	21:00–22:00	22:00–23:00	23:00–24:00
Baseline	0.6204 (0.01)	0.6209 (0.01)	0.616 (0.01)	0.6188 (0.01)	0.6179 (0.01)
Sleep Restriction 1	0.6178 (0.01)	0.6198 (0.01)	0.6214 (0.01)	0.6182 (0.01)	0.6171 (0.01)
Sleep Restriction 3	0.62 (0.01)	0.6209 (0.01)	0.6204 (0.01)	0.6119 (0.01)	0.6228 (0.01)
Sleep Restriction 5	0.6288 (0.01)	0.627 (0.01)	0.6248 (0.01)	0.6273 (0.01)	0.6236 (0.01)
Sleep Recovery 1	0.6238 (0.01)	0.6264 (0.01)	0.6237 (0.01)	0.6243 (0.01)	0.6288 (0.01)
Sleep Recovery 3	0.6199 (0.01)	0.6221 (0.01)	0.6212 (0.01)	0.6203 (0.01)	0.6227 (0.01)

*Note:* Table 6A–D contains the descriptive statistics of Spectral Intercept in WAKE, NREM, REM episodes, and when all scores were considered (ALL), in the following format: mean (standard deviation).

## 5 Discussion

### 5.1 Spectral Slope, Spectral Intercept and Normalised Spectral Entropy

The present study investigates the sleep deprivation‐related neurophysiological signals (electrocorticography, ECoG) of homeostatic adjustments during sleep, using the classical and a novel, data‐driven signal processing method, and an information theoretical approach. The spectral slope (the steepness of the log–log power spectrum) compared to SWA (0.75–4.5 Hz), better captures the effects of prolonged wakefulness on sleep homeostasis. More precisely, suggesting the robustness of the spectral slope, it captures the increase of sleep deprivation‐related enhanced sleep depth during both REM and NREM phases, as opposed to SWA only showing sleep homeostatic adjustments during the NREM phase; in addition, the spectral slope reflects on sleep homeostasis outside the frequency range of what is traditionally considered a biomarker of sleep depth. In other words, the spectral slope appears to be a more composite index of sleep depth than the SWA. In addition, the spectral intercept, that is the point at which the model touches the ordinate‐axis on the log–log transformed plane, reflects on sleep deprivation‐related homeostatic processes during REM and NREM, just as the spectral slope does. This is easily justified by the correlation between the spectral slope and the spectral intercept (Bódizs et al. 2021). Finally, the results suggest that normalized spectral entropy fails to differentiate between baseline sleep and sleep following sleep deprivation in mice. Although entropy measures are highly correlated with aperiodic parameters (Höhn et al. 2024), entropy‐based indices tend to require large sample sizes for accurate assessment of information content (Borst and Theunissen 1999; Chao et al. 2013; Liu et al. 2016; Manis et al. 2018). The absence of the expected correlations does not necessarily contradict previous findings, but may reflect sampling constraints specific to the current dataset (*N* = 8).

The interpretation of the spectral slope is, however, a non‐trivial matter. The surface neurophysiological power spectrum follows a tail‐heavy exponential distribution, which upon a double logarithmic transformation linearizes, enabling the fit of a first‐degree polynomial. As the domain is the log frequency bins, the slope of the linear curve can be considered reflecting on the ratio of slow oscillations to fast oscillations; in which case a steeper curve, that is higher absolute values of the spectral slope, means that low‐frequency oscillations are relatively more dominant compared to high‐frequency oscillations. On the other hand, a flat curve reveals the enhancement of fast oscillations. Therefore, in the context of sleep homeostasis, the steepness of the curve fitted over the power spectrum is, at heart, an expression of sleep depth (Bódizs et al. 2021). The Fractal and Oscillatory Adjustment Model (FOAM; Bódizs et al. 2024) is a theoretical framework (a response to the scientific results aggregating since the conception of the two‐process model) integrating homeostatic sleep pressure, circadian rhythms, and ultradian cycles into a comprehensive model. That is to say, it integrates the concept of fractal‐like patterns with oscillatory processes to explain how the regulation of the sleep–wake cycle is achieved. In the context of FOAM, the spectral slope is a key parameter characterising the scaling properties of the brain. Specifically, it reflects the fractal nature of neural background activity and sleep homeostasis. Therefore, the spectral slope may change in response to sleep pressure, making it a marker of sleep–wake history.

Furthermore, the spectral slope can be understood in terms of critical behaviour. Criticality, in essence, is a macroscopic feature that is the product of a balance between micro‐level processes of the states in which a system can exist. Said balance corresponds to the phase transition between these states, and therefore, criticality is a characteristic of phase transitions, at which new properties emerge (Beggs and Timme 2012; Plenz et al. 2021; Zimmern 2020)—in terms of the brain, these properties manifest themselves in optimal computing, such as long‐range communication or maximal information transfer between neuronal populations. Systems expressing scale‐invariant phenomena may be characterised by criticality (Bak and Creutz 1994). Critical dynamics display scale invariance because at the critical point (or Griffiths phase, assuming a quasi‐critical system), the system lacks any characteristic length or time scale (for a more in‐depth explanation, see Appendix A). This means that fluctuations occur on all possible sizes and timescales. In such states, microscopic interactions cascade into macroscopic effects, leading to power‐law distributions. This behaviour reflects the fractal nature of critical phenomena: zooming in or out reveals self‐similar structures. In the context of self‐organised criticality, systems naturally tune themselves to this critical point, perpetually residing in a marginally stable state where any small perturbation can trigger chain reactions of all sizes; a hallmark of scale‐invariant dynamics.

Building on this, it is essential to clarify that the scaling exponent does not only indicate a static critical state, but rather reflects critical dynamics—that is, how the system evolves in proximity to criticality and shifts towards persistent and anti‐persistent states. Thus, the power‐law scaling exponent (spectral slope) indexing fractal behaviour can be considered an index of critical dynamics. Investigating criticality involves the identification of an order and a control parameter. The order parameter is the macroscopic property characteristic of a given phase, while changes in the control parameter drive the fluctuations of the order parameter (Zimmern 2020). In the context of the present study, the order parameter is the spectral slope, and the control parameter is the homeostatic pressure (induced by the experimental manipulation: baseline sleep vs. sleep following prolonged wakefulness). Wakefulness is speculated to drive the brain towards supercritical behaviour, resulting in continuously flattening power spectra (Pearlmutter and Houghton 2009). Therefore, one may infer that sleep deprivation, that is inappropriately prolonged wakefulness, should exacerbate this phenomenon. The organic consequence of such is elevated sleep pressure resulting in a need for compensation, and thus a more pronounced movement towards subcriticality should be apparent in order for the system (the brain) to self‐organise back into criticality. In other words, recovery sleep following sleep deprivation should bear signatures of pronounced subcriticality, such as a steeper power spectrum in the log–log space.

From an oscillatory point of view, supercriticality corresponds to a flat power spectrum, also called white noise, while subcriticality corresponds to a 1/*f* ^2^ power spectrum, also called brown noise (Ros et al. 2014). Curiously, in between the flat white noise and the fast decaying brown noise lies the hyperbolic function in the form of 1/*f*, also called pink noise, which happens to be a signature fractal process of complex systems maintaining criticality possibly by a mechanism denoted self‐organised criticality (Bak et al. 1987). In light of this framework, the steepened power spectra observed during recovery sleep may indicate a compensatory shift towards subcriticality, counteracting the presumed supercritical drift induced by prolonged wakefulness. While our findings align with this directional interpretation, prior empirical work suggests that −2 may act as an attractor exponent, a value to which a complex system tends to self‐organise into. This exponent characterises the Brownian‐type power spectrum, and typically appears when rapid cortical activity and inactivity alternate in the nervous system (Bódizs et al. 2024). Thus, a lower spectral exponent reflects a steeper spectral slope and the tendency for subcriticality, whereas a higher (or more positive) spectral exponent indicates the tendency towards supercriticality. We note that the observed spectral exponents (see Table 4A–D) are steeper than what is typically expected for a system near criticality.

This discrepancy, however, may be justified by the shift of the spectral knee towards higher frequencies (~9 Hz) rendering the scale‐free portion of the power spectrum to decay faster and resulting in scaling exponents below the expected value. In addition, the brain's tendency to organise into a subcritical regime and the relative simplicity of the rodent brain may also serve as arguments in favour of presenting our results in terms of the critical brain hypothesis. It has been previously suggested that the brain operates at a slightly subcritical regime during normal wake activity (Meisel et al. 2013; Pearlmutter and Houghton 2009), also supported by human intracranial recordings of neuronal avalanches (Priesemann et al. 2013). Existing in a somewhat subcritical state allows the brain to avoid excessive noise in neural networks. Although near criticality offers maximal flexibility and responsiveness, a slightly subcritical regime provides a balance between stability and flexibility, minimising the risk of runaway excitations or chaotic behaviour, and ensuring that neural responses are more stable and predictable. Moreover, the complexity of neural dynamics plays a significant role in whether the brain can operate near criticality or whether it is constrained to function in a suboptimal state. Rodent brains have simpler neural architecture, with fewer neurons and less intricate connectivity patterns as compared to human brains (Kaas and Herculano‐Houzel 2017; Van Essen et al. 2019). The reduced complexity may limit the ability of rodent brains to exhibit the neural coordination necessary for criticality. In addition, the cognitive demands on a rodent are generally less diverse and complex than those on a human. Rodent brains are optimised for survival tasks like navigating, foraging, and escaping predators. These tasks require more ordered, predictable, stable neural dynamics that favour subcritical states—operating in a subcritical regime helps minimise metabolic costs. While energy efficiency is important in the human brain, its larger metabolic budget allows for more neural variability and activity. This increased metabolic capacity may support long‐range synchrony, information integration, and flexibility, allowing the brain to explore a wider range of states. Thus, it can afford the energy costs of operating closer to criticality while still balancing metabolic demands.

### 5.2 Spectral Knee: Allometric Scaling Hypothesis

The spectral knee, defined as the transition point in the power spectrum from low‐frequency (white noise‐like) to high‐frequency (power‐law or coloured noise) activity, appears markedly shifted towards higher frequencies in the mouse brain (~9–10 Hz). This is notably above the conventional SWA range (0.75–4.5 Hz) typically associated with sleep homeostasis. Despite this shift, the spectral slope remains predictive of homeostatic sleep pressure, suggesting that the underlying neurophysiological markers may scale across species in ways not fully captured by traditional frequency bands.

We propose that this spectral shift is a structural property, and it is a consequence of finite‐size effects: smaller brains impose physical limits on the extent of neuronal synchrony, especially at slow oscillations. Large‐scale oscillations (such as slow waves) require extensive neural mass and long conduction pathways to achieve global coordination (Nunez and Srinivasan 2014; Sanchez‐Vives 2020). In smaller animals like mice, the anatomical constraints may reduce the maximum achievable correlation length, thereby compressing the range over which scale‐free, fractal‐like dynamics can emerge. As a result, the transition between global and local regimes, that is the spectral knee, possibly occurs at a higher frequency.

This interpretation aligns with principles from finite‐size scaling (FSS) in statistical physics, where systems near a critical point exhibit critical‐like coordination only up to a size‐dependent limit (Ardourel and Bangu 2023). In finite systems, criticality is ‘rounded’, meaning power‐law behaviour is truncated by system boundaries. The spectral knee, in this context, may be viewed as a biological signature of such finite‐size criticality.

To formalise this idea, we draw from allometric scaling principles, which describe how biological traits scale with body size (Mchanon and Bonner 1983; Schmidt‐Nielsen 1984). If the spectral knee (*Y*) scales with body or brain mass (*M*) according to a power law of the form:

(7) $$Y=Y_{0}M^{b}$$

then a log–log transformation yields a linear relationship:

(8) $$\log\left(Y\right)=\log\left(Y_{0}\right)+b\times \log\left(M\right)$$

Furthermore, the Matérn process offers a flexible model for interpreting these spectral features (Lilly et al. 2017). Unlike fractional Brownian motion, the Matérn process introduces a plateau at low frequencies, capturing finite‐range correlations and reflecting the physical limits of hierarchical or turbulent systems. In the frequency domain, this appears as a broken power‐law, with white noise at the lowest frequencies, coloured noise at higher frequencies, and a transition (the spectral knee) between the two. This plateau may thus signify the inability of the system to sustain global coherence beyond a certain scale—a hallmark of finite‐size effects and a potential indicator of critical dynamics constrained by anatomical structure.

Taken together, these ideas suggest that the spectral knee represents a neurophysiological boundary condition shaped by anatomical scale. In smaller brains, this limit is reached at higher frequencies, flattening low‐frequency power and shifting fractal‐like behaviour into a compressed spectral window. Recognising the spectral knee as a finite‐size marker may offer new insights into how sleep homeostasis and large‐scale brain dynamics are constrained by the physical architecture of the brain.

### 5.3 Spectral Knee: Neuronal Timescale Model

In addition, the spectral knee can be understood as marking a characteristic neuronal time scale, where knee frequency inversely maps to a network time constant: the characteristic duration over which neural activity persists or remains correlated in time. That is, it reflects how long a neuron or neural population integrates information before its activity decays or resets. Gao et al. (2020) demonstrated that by parameterizing the aperiodic component of intracranial recordings, the knee frequency (*f*_k_) yields a decay constant *τ* = 1/(2π*f*_k_), representing how long population‐level activity persists before decorrelating. High knee frequencies thus correspond to shorter time constants (fast fluctuations), while lower knee frequencies indicate longer‐lasting dynamics. Importantly, multiple distinct knees have been observed: for example, at ~1–5 Hz (∼30–160 ms), ~20–45 Hz (∼4–8 ms), and ~65–75 Hz (∼2–3 ms); each likely reflecting different physiological processes, from slow synaptic or network interactions to fast receptor dynamics.

While the interpretation of the spectral knee as a marker of neuronal timescales is conceptually appealing, this model rests on a critical assumption that the spectral exponent *α* = 2, corresponding to a Lorentzian power spectrum and a single exponential decay in the time domain. However, in our data, the observed spectral slopes deviate from this condition, often ranging between 2.5 and 3.5 depending on brain state. When α ≠ 2, the knee frequency no longer maps linearly onto a single characteristic timescale, rendering the *τ* = 1/(2π*f*_k_) transformation mathematically invalid. Moreover, changes in the spectral knee may co‐occur with changes in slope, confounding any straightforward interpretation in terms of neural processing speed. Given these limitations and considering that our data span across neurophysiologically distinct states like wakefulness, NREM, and REM we caution against the literal application of the Gao model. Instead, we treat the timescale interpretation as one of several complementary perspectives, rather than a definitive mechanistic explanation.

Additionally, previous literature reports longer neuronal processing timescales during sleep compared to wakefulness (Lendner et al. 2023), and that deep sleep (N3) is associated with the longest processing time (Ameen et al. 2024); consistent with the idea of extended temporal integration in low‐arousal states. However, in our data, we observe higher spectral knee frequencies during REM and NREM sleep relative to wakefulness, implying longer processing timescales when awake compared to sleeping: an interpretation that runs counter to these prior findings. This discrepancy likely arises from fundamental limitations in applying the timescale model across distinct brain states, especially given the assumption of the model regarding the spectral exponent, which is violated in our data. Moreover, the co‐variation of spectral slope and knee frequency complicates attributing knee shifts solely to changes in processing speed, since slope changes themselves alter spectral shape and could reflect differences in neural synchrony or excitation rather than timescale per se. Adding further complexity, we find that sleep deprivation‐related modulation of the spectral knee occurs exclusively during REM sleep. This REM‐specific effect remains unexplained within both the timescale model and our brain‐size hypothesis, highlighting an important gap in understanding the functional significance of the spectral knee across sleep states. Addressing these will require focused experiments probing behavioural and cognitive correlates of spectral knee dynamics during REM, which lie beyond the current study.

The timescale model likely captures important functional aspects of neural dynamics, particularly the temporal integration capacity of cortical circuits during different cognitive or arousal states. In contrast, our hypothesis focuses on structural constraints, proposing that the spectral knee reflects anatomical limitations on large‐scale synchronisation imposed by brain size (see Section 6.2). Rather than viewing these perspectives as competing, we consider them complementary: the functional timescale framework describes how rapidly neural populations process information, while our structural account addresses how system size shapes the frequency range over which coherent dynamics can emerge. Together, these models offer a more complete understanding of the spectral knee, linking both transient computational processes and stable anatomical features.

### 5.4 Limitations: Stress Induced by Prolonged Wakefulness

We acknowledge that the use of 18 h of sleep deprivation via a rotating wheel, while effective in maintaining wakefulness, may introduce significant stress to the animals. Previous studies have shown that such prolonged deprivation protocols can elevate corticosterone levels by a factor of 3–4 (Dispersyn et al. 2017; Roman et al. 2006), indicating a marked activation of the hypothalamic–pituitary–adrenal (HPA) axis. This stress response could potentially confound the interpretation of neurophysiological changes attributed to sleep homeostatic processes; that is to say, elevated stress levels may independently influence cortical excitability, oscillatory dynamics, and spectral characteristics, including the spectral slope and entropy measures assessed in this study. While our primary objective was to evaluate neural signatures of sleep pressure, we recognize that the observed effects may be partially modulated by stress‐related mechanisms, and this should be considered when interpreting the findings. Future studies employing less invasive or shorter‐duration deprivation protocols, combined with corticosterone assays, would help disentangle the effects of sleep loss from stress‐related neural alterations.

## 6 Conclusion

We found that the fractal parameters (spectral slope and spectral intercept) of the neurophysiological power spectrum have better performance in capturing sleep deprivation‐related homeostatic adjustments compared to the classical SWA approach. This is apparent as the fractal parameters reflect on sleep homeostasis irrespective of sleep staging; that is, sleep deprivation is indexed during both REM and NREM. Moreover, the shift of the spectral knee towards higher frequencies (~9–10 Hz) renders the spectral slope unreflective of SWA, which is intriguing considering the history of SWA in the study of sleep homeostasis.

## Author Contributions

**Tárek Zoltán Magyar:** conceptualization, formal analysis, investigation, writing – original draft. **Róbert Bódizs:** conceptualization, formal analysis, investigation, supervision, writing – review and editing. **Orsolya Szalárdy:** supervision, writing – review and editing.

## Conflicts of Interest

The authors declare no conflicts of interest.

## Supporting information


**Data S1:** jsr70232‐sup‐0001‐supinfo.docx.

## Data Availability

The data that support the findings of this study are openly available in GIN at https://gin.g‐node.org/hiobeen/Mouse_EEG_ChronicSleepRestriction_Kim_et_al.

## Appendix A

An explanation of why criticality exhibits scale invariance lies in the way correlations propagate and dominate at critical points in physical systems: **(1) Diverging correlation length:** At a critical point (e.g., in a phase transition), the correlation length (ξ; the typical distance over which parts of the system influence each other) diverges. This means there is no characteristic length scale governing the behavior of the system. **(2) Coupling of all scales:** Because ξ → ∞, fluctuations at all spatial and temporal scales are equally relevant. Tiny perturbations can affect both small and large regions. This implies that the system cannot be described by localized or isolated behaviors; that is, it must be treated as a whole. **(3) Emergence of self‐similarity:** With all length scales participating, self‐similar patterns emerge. Observing the system at different magnifications reveals similar structures, a hallmark of fractal geometry. For example, in percolation clusters or sandpile avalanches, small and large events follow the same rules and appearance. **(4) Power law distributions:** Because there's no typical event size, the distribution of event sizes (like avalanches in self‐organized criticality or domain sizes in magnetism) follows a power law, which mathematically encodes scale invariance. That is to say, rescaling the variable does not change the form of the distribution.

At its core, the correlation length ξ describes how far one part of a system influences another. It is the typical distance over which fluctuations in one region are statistically correlated with another. In non‐critical systems, ξ is finite: distant parts of the system are uncorrelated. However, at a critical point, ξ → ∞: correlations do not decay exponentially, and no finite distance separates independent regions, that is, everything is interconnected.

The divergence follows a power‐law behavior near the critical point:

(1A)
$$\xi ∼{\left|T-T_{C}\right|}^{-v}$$

where ξ is the correlation length, T is the control parameter (e.g., temperature), Tc is the critical temperature (or critical point), ν is a critical exponent, characteristic of the universality class of the system. This equation says: As the system approaches the critical point T → Tc, the denominator goes to zero, and so ξ → ∞. The rate of divergence (Diverge means a value does not settle at any finite number; in other words, it grows without bound; Rate of divergence means how quickly (controlled by ν) the correlation length ξ grows (or diverges) as the control parameter T approaches the critical point Tc) is governed by the exponent ν, which varies by system type.

**TABLE A1:** Post hoc tests of the interaction between DAYS and HOURS of SWA during NREM.

		*t*	*p*_bonf_	*d*
SR1, 19–20	R1, 20–21	4.577	0.008	1.346
	R5, 23–24	4.807	0.003	1.414
SR3, 19–20	R1, 20–21	5.018	0.002	1.476
	SR3, 23–24	4.971	< 0.001	0.925
	R1, 23–24	5.248	< 0.001	1.543
SR5, 19–20	R1, 23–24	4.646	0.006	1.366
SR3, 20–21	R1, 23–24	4.474	0.012	1.316

**TABLE A2:** Post hoc tests of the interaction between DAYS and HOURS of spectral intercept during NREM.

		*t*	*p*_bonf_	*d*
SR1, 19–20	R1, 19–20	6.308	< 0.001	1.575
	R1, 20–21	6.411	< 0.001	1.626
	R3, 20–21	4.431	0.013	1.123
	R1, 21–22	5.528	< 0.001	1.402
	R3, 21–22	4.519	0.009	1.146
	R1, 22–23	5.149	< 0.001	1.306
	R3, 22–23	4.851	0.003	1.231
	R1, 23–24	5.348	< 0.001	1.356
	R3, 23–24	4.533	0.009	1.1493
SR3, 19–20	R1, 19–20	5.241	< 0.001	1.311
	R1, 20–21	5.369	< 0.001	1.362
	R1, 21–22	4.487	0.011	1.138
SR5, 19–20	R1, 19–20	6.179	< 0.001	1.542
	R1, 20–21	6.283	< 0.001	1.593
	R1, 21–22	5.401	< 0.001	1.369
	SR5, 22–23	4.502	0.005	0.752
	R1, 22–23	5.021	0.002	1.273
	R3, 22–23	4.723	0.004	1.198
	R1, 23–24	5.221	< 0.001	1.324
R1, 19–20	SR1, 20–21	−5.181	< 0.001	−1.314
	SR3, 20–21	−5.325	< 0.001	−1.351
	SR5, 20–21	−5.006	0.002	−1.269
	SR1, 21–22	−4.553	0.008	−1.155
SR1, 20–21	R1, 20–21	5.468	< 0.001	1.365
	R1, 21–22	4.499	0.011	1.141
SR3, 20–21	R1, 20–21	5.615	< 0.001	1.401
	R1, 21–22	4.644	0.006	1.177
	R1, 23–24	4.464	0.011	1.132
SR5, 20–21	R1, 20–21	5.291	< 0.001	1.321
R1, 20–21	SR1, 21–22	−4.755	0.004	−1.206
	SR5, 21–22	−4.599	0.007	−1.166

**TABLE A3:** Post hoc tests of the interaction between DAYS and HOURS of spectral slope during REM.

		*t*	*p*_bonf_	*d*
SR1, 19–20	R1, 20–21	4.961	0.002	1.112
SR3, 19–20	R1, 20–21	5.211	< 0.001	1.166
	R1, 23–24	4.969	0.002	1.112
SR5, 19–20	R1, 19–20	4.815	0.003	1.103
	R1, 20–21	5.616	< 0.001	1.257
	R1, 21–22	4.848	0.003	1.085
	SR5, 23–24	4.521	0.008	0.802
	R1, 23–24	5.373	< 0.001	1.202
R1, 19–20	SR5, 20–21	−4.535	0.011	−1.015
SR1, 20–21	R1, 20–21	4.909	0.002	1.124
	R1, 23–24	4.782	0.004	1.071
SR5, 20–21	R1, 20–21	5.101	0.002	1.168
	R1, 23–24	4.978	0.002	1.114

**TABLE A4:** Post hoc tests of the interaction between DAYS and HOURS of spectral slope during NREM.

		*t*	*p*_bonf_	*d*
SR1, 19–20	R1, 19–20	7.1739	1.6667	< 0.001
	R3, 19–20	4.9289	1.1451	0.002
	R1, 20–21	6.9596	1.6456	< 0.001
	R3, 20–21	4.8204	1.1398	0.003
	R1, 21–22	6.2613	1.4805	< 0.001
	R3, 21–22	4.8487	1.1465	0.003
	R1, 22–23	5.6941	1.3464	< 0.001
	R3, 22–23	5.1313	1.2133	< 0.001
	R1, 23–24	5.7895	1.3689	< 0.001
	R3, 23–24	4.8184	1.1393	0.003
SR3, 19–20	R1, 19–20	5.8612	1.3617	< 0.001
	R1, 20–21	5.6697	1.3406	< 0.001
	R1, 21–22	4.9715	1.1755	0.002
	R1, 23–24	4.4997	1.064	0.011
SR5, 19–20	R1, 19–20	6.3984	1.4865	< 0.001
	R1, 20–21	6.1975	1.4654	< 0.001
	R1, 21–22	5.4993	1.3003	< 0.001
	R1, 22–23	4.9321	1.1662	0.002
	R1, 23–24	5.0275	1.1888	0.002
R1, 19–20	SR1, 20–21	−5.9425	−1.4051	< 0.001
	SR3, 20–21	−5.9525	−1.4075	< 0.001
	SR5, 20–21	−5.5589	−1.3144	< 0.001
	SR1, 21–22	−5.2077	−1.2314	< 0.001
	SR3, 21–22	−4.9139	−1.1619	0.002
	SR5, 21–22	−4.7575	−1.1249	0.003
	SR1, 22–23	−4.4317	−1.0479	0.012
	SR1, 23–24	−4.4565	−1.0537	0.011
SR1, 20–21	R1, 20–21	5.957	1.384	< 0.001
	R1, 21–22	5.1549	1.2189	< 0.001
	R1, 22–23	4.5877	1.0848	0.006
	R1, 23–24	4.6831	1.1073	0.004
SR3, 20–21	R1, 20–21	5.9671	1.3863	< 0.001
	R1, 21–22	5.1649	1.2212	< 0.001
	R1, 22–23	4.5977	1.0871	0.006
	R1, 23–24	4.6931	1.1097	0.004
SR5, 20–21	R1, 20–21	5.5666	1.2933	< 0.001
	R1, 21–22	4.7714	1.1282	0.003
	SR1, 21–22	−5.1184	−1.2102	< 0.001
	SR3, 21–22	−4.8246	−1.1408	0.002
	SR5, 21–22	−4.6681	−1.1038	0.004
	R1, 21–22	4.4986	1.0451	0.01
R1, 20–21	SR1, 21–22	−5.1184	−1.2102	< 0.001
	SR3, 21–22	−4.8246	−1.1408	0.002
	SR5, 21–22	−4.6681	−1.1038	0.004
SR1, 21–22	R1, 21–22	4.4986	1.0451	0.011

## References

[jsr70232-bib-0001] Ameen, M. S. , J. Jacobs , M. Schabus , K. Hoedlmoser , and T. Donoghue . 2024. “The Temporal Dynamics of Aperiodic Neural Activity Track Changes in Sleep Architecture.” In bioRxiv. Cold Spring Harbor Laboratory. 10.1101/2024.01.25.577204.

[jsr70232-bib-0002] Ardourel, V. , and S. Bangu . 2023. “Finite‐Size Scaling Theory: Quantitative and Qualitative Approaches to Critical Phenomena.” Studies in History and Philosophy of Science 100: 99–106. 10.1016/j.shpsa.2023.05.010. 37379613

[jsr70232-bib-0003] Bak, P. , and M. Creutz . 1994. “Fractals and Self‐Organized Criticality.” In Fractals in Science, edited by A. Bunde and S. Havlin , 27–48. Springer. 10.1007/978-3-642-77953-4_2.

[jsr70232-bib-0004] Bak, P. , C. Tang , and K. Wiesenfeld . 1987. “Self‐Organized Criticality: An Explanation of the 1/f Noise.” Physical Review Letters 59, no. 4: 381–384. 10.1103/PhysRevLett.59.381. 10035754

[jsr70232-bib-0005] Beggs, J. , and N. Timme . 2012. “Being Critical of Criticality in the Brain.” Frontiers in Physiology 3. 10.3389/fphys.2012.00163. PMC3369250 22701101

[jsr70232-bib-0006] Beggs, J. M. , and D. Plenz . 2003. “Neuronal Avalanches in Neocortical Circuits.” Journal of Neuroscience: The Official Journal of the Society for Neuroscience 23, no. 35: 11167–11177. 10.1523/JNEUROSCI.23-35-11167.2003. 14657176 PMC6741045

[jsr70232-bib-0007] Bódizs, R. , B. Schneider , P. P. Ujma , C. G. Horváth , M. Dresler , and Y. Rosenblum . 2024. “Fundamentals of Sleep Regulation: Model and Benchmark Values for Fractal and Oscillatory Neurodynamics.” Progress in Neurobiology 234: 102589. 10.1016/j.pneurobio.2024.102589. 38458483

[jsr70232-bib-0008] Bódizs, R. , O. Szalárdy , C. Horváth , et al. 2021. “A Set of Composite, Non‐Redundant EEG Measures of NREM Sleep Based on the Power Law Scaling of the Fourier Spectrum.” Scientific Reports 11, no. 1: 2041. 10.1038/s41598-021-81230-7. 33479280 PMC7820008

[jsr70232-bib-0009] Borbély, A. A. 1982. “A Two Process Model of Sleep Regulation.” Human Neurobiology 1, no. 3: 195–204. 7185792

[jsr70232-bib-0010] Borbély, A. A. , S. Daan , A. Wirz‐Justice , and T. Deboer . 2016. “The Two‐Process Model of Sleep Regulation: A Reappraisal.” Journal of Sleep Research 25, no. 2: 131–143. 10.1111/jsr.12371. 26762182

[jsr70232-bib-0011] Borst, A. , and F. E. Theunissen . 1999. “Information Theory and Neural Coding.” Nature Neuroscience 2, no. 11: 11. 10.1038/14731. 10526332

[jsr70232-bib-0012] Chao, A. , Y. T. Wang , and L. Jost . 2013. “Entropy and the Species Accumulation Curve: A Novel Entropy Estimator via Discovery Rates of New Species.” Methods in Ecology and Evolution 4, no. 11: 1091–1100. 10.1111/2041-210X.12108.

[jsr70232-bib-0013] Chialvo, D. R. 2010. “Emergent Complex Neural Dynamics.” Nature Physics 6, no. 10: 10. 10.1038/nphys1803.

[jsr70232-bib-0014] Dispersyn, G. , F. Sauvet , D. Gomez‐Merino , et al. 2017. “The Homeostatic and Circadian Sleep Recovery Responses After Total Sleep Deprivation in Mice.” Journal of Sleep Research 26, no. 5: 531–538. 10.1111/jsr.12541. 28425172

[jsr70232-bib-0015] Donoghue, T. , M. Haller , E. J. Peterson , et al. 2020. “Parameterizing Neural Power Spectra Into Periodic and Aperiodic Components.” Nature Neuroscience 23, no. 12: 1655–1665. 10.1038/s41593-020-00744-x. 33230329 PMC8106550

[jsr70232-bib-0016] Flood, M. W. , and B. Grimm . 2021. “EntropyHub: An Open‐Source Toolkit for Entropic Time Series Analysis.” PLoS One 16, no. 11: e0259448. 10.1371/journal.pone.0259448. 34735497 PMC8568273

[jsr70232-bib-0017] Gao, R. , E. J. Peterson , and B. Voytek . 2017. “Inferring Synaptic Excitation/Inhibition Balance From Field Potentials.” NeuroImage 158: 70–78. 10.1016/j.neuroimage.2017.06.078. 28676297

[jsr70232-bib-0018] Gao, R. , R. L. van den Brink , T. Pfeffer , and B. Voytek . 2020. “Neuronal Timescales Are Functionally Dynamic and Shaped by Cortical Microarchitecture.” eLife 9: e61277. 10.7554/eLife.61277. 33226336 PMC7755395

[jsr70232-bib-0019] Han, H.‐B. , B. Kim , Y. Kim , Y. Jeong , and J. H. Choi . 2022. “Nine‐Day Continuous Recording of EEG and 2‐Hour of High‐Density EEG Under Chronic Sleep Restriction in Mice.” Scientific Data 9, no. 1: 225. 10.1038/s41597-022-01354-x. 35606461 PMC9126869

[jsr70232-bib-0021] He, B. J. 2014. “Scale‐Free Brain Activity: Past, Present, and Future.” Trends in Cognitive Sciences 18, no. 9: 480–487. 10.1016/j.tics.2014.04.003. 24788139 PMC4149861

[jsr70232-bib-0022] Helakari, H. , M. Järvelä , T. Väyrynen , et al. 2023. “Effect of Sleep Deprivation and NREM Sleep Stage on Physiological Brain Pulsations.” Frontiers in Neuroscience 17: 1275184. 10.3389/fnins.2023.1275184. 38105924 PMC10722275

[jsr70232-bib-0023] Höhn, C. , M. A. Hahn , J. D. Lendner , and K. Hoedlmoser . 2024. “Spectral Slope and Lempel–Ziv Complexity as Robust Markers of Brain States During Sleep and Wakefulness.” ENeuro 11, no. 3: ENEURO.0259‐23.2024. 10.1523/ENEURO.0259-23.2024. PMC10978822 38471778

[jsr70232-bib-0024] Horváth, G. , C. Szalárdy , O. P. P. Ujma , et al. 2022. “Overnight Dynamics in Scale‐Free and Oscillatory Spectral Parameters of NREM Sleep EEG.” Scientific Reports 12, no. 1: 1. 10.1038/s41598-022-23033-y. 36319742 PMC9626458

[jsr70232-bib-0025] Horváth, G. C. , and R. Bódizs . 2025. “Effect of Sleep Deprivation on Fractal and Oscillatory Spectral Measures of the Sleep EEG: A Window on Basic Regulatory Processes.” NeuroImage 314: 121260. 10.1016/j.neuroimage.2025.121260. 40349742

[jsr70232-bib-0026] Hou, F. , Z. Yu , C.‐K. Peng , A. Yang , C. Wu , and Y. Ma . 2018. “Complexity of Wake Electroencephalography Correlates With Slow Wave Activity After Sleep Onset.” Frontiers in Neuroscience 12: 809. 10.3389/fnins.2018.00809. 30483046 PMC6243118

[jsr70232-bib-0027] Inouye, T. , K. Shinosaki , H. Sakamoto , et al. 1991. “Quantification of EEG Irregularity by Use of the Entropy of the Power Spectrum.” Electroencephalography and Clinical Neurophysiology 79, no. 3: 204–210. 10.1016/0013-4694(91)90138-T. 1714811

[jsr70232-bib-0028] Kaas, J. H. , and S. Herculano‐Houzel . 2017. “What Makes the Human Brain Special: Key Features of Brain and Neocortex.” In The Physics of the Mind and Brain Disorders: Integrated Neural Circuits Supporting the Emergence of Mind, edited by I. Opris and M. F. Casanova , 3–22. Springer International Publishing. 10.1007/978-3-319-29674-6_1.

[jsr70232-bib-1101] Keshmiri, S. 2020. “Entropy and the Brain: An Overview.” Entropy 22, no. 9: 917. 10.3390/e22090917. 33286686 PMC7597158

[jsr70232-bib-0029] Kim, B. , E. Hwang , R. E. Strecker , J. H. Choi , and Y. Kim . 2020. “Differential Modulation of NREM Sleep Regulation and EEG Topography by Chronic Sleep Restriction in Mice.” Scientific Reports 10, no. 1: 18. 10.1038/s41598-019-54790-y. 31924847 PMC6954245

[jsr70232-bib-0030] Lendner, J. D. , R. F. Helfrich , B. A. Mander , et al. 2020. “An Electrophysiological Marker of Arousal Level in Humans.” eLife 9: e55092. 10.7554/eLife.55092. 32720644 PMC7394547

[jsr70232-bib-0032] Lendner, J. D. , N. Niethard , B. A. Mander , et al. 2023. “Human REM Sleep Recalibrates Neural Activity in Support of Memory Formation.” Science Advances 9, no. 34: eadj1895. 10.1126/sciadv.adj1895. 37624898 PMC10456851

[jsr70232-bib-0054] Lilly, J. M. , A. M. Sykulski , J. J. Early , and S. C. Olhede . 2017. “Fractional Brownian Motion, the Matérn Process, and Stochastic Modeling of Turbulent Dispersion.” Nonlinear Processes in Geophysics 24, no. 3: 481–514. 10.5194/npg-24-481-2017.

[jsr70232-bib-0033] Liu, D. , D. Wang , Y. Wang , et al. 2016. “Entropy of Hydrological Systems Under Small Samples: Uncertainty and Variability.” Journal of Hydrology 532: 163–176. 10.1016/j.jhydrol.2015.11.019.

[jsr70232-bib-0034] Manis, G. , M. Aktaruzzaman , and R. Sassi . 2018. “Low Computational Cost for Sample Entropy.” Entropy 20, no. 1: 1. 10.3390/e20010061. PMC7512258 33265148

[jsr70232-bib-0035] Mchanon, T. , and J. T. Bonner . 1983. On Size and Life. Scientific American Books.

[jsr70232-bib-0036] Meisel, C. , E. Olbrich , O. Shriki , and P. Achermann . 2013. “Fading Signatures of Critical Brain Dynamics During Sustained Wakefulness in Humans.” Journal of Neuroscience 33, no. 44: 17363–17372. 10.1523/JNEUROSCI.1516-13.2013. 24174669 PMC3858643

[jsr70232-bib-0038] Nunez, P. L. , and R. Srinivasan . 2014. “Neocortical Dynamics due to Axon Propagation Delays in Cortico‐Cortical Fibers: EEG Traveling and Standing Waves With Implications for Top‐Down Influences on Local Networks and White Matter Disease.” Brain Research 1542: 138–166. 10.1016/j.brainres.2013.10.036. 24505628 PMC3942804

[jsr70232-bib-0039] Pearlmutter, B. A. , and C. J. Houghton . 2009. “A New Hypothesis for Sleep: Tuning for Criticality.” Neural Computation 21, no. 6: 1622–1641. 10.1162/neco.2009.05-08-787. 19191602

[jsr70232-bib-0040] Plenz, D. , T. L. Ribeiro , S. R. Miller , P. A. Kells , A. Vakili , and E. L. Capek . 2021. “Self‐Organized Criticality in the Brain.” Frontiers in Physics 9: 639389. https://www.frontiersin.org/articles/10.3389/fphy.2021.639389.

[jsr70232-bib-0041] Priesemann, V. , M. Valderrama , M. Wibral , and M. Le Van Quyen . 2013. “Neuronal Avalanches Differ From Wakefulness to Deep Sleep—Evidence From Intracranial Depth Recordings in Humans.” PLoS Computational Biology 9, no. 3: e1002985. 10.1371/journal.pcbi.1002985. 23555220 PMC3605058

[jsr70232-bib-0042] Roman, V. , R. Hagewoud , P. G. M. Luiten , and P. Meerlo . 2006. “Differential Effects of Chronic Partial Sleep Deprivation and Stress on Serotonin‐1A and Muscarinic Acetylcholine Receptor Sensitivity.” Journal of Sleep Research 15, no. 4: 386–394. 10.1111/j.1365-2869.2006.00555.x. 17118095

[jsr70232-bib-0043] Ros, T. , J. B. Baars , R. A. Lanius , and P. Vuilleumier . 2014. “Tuning Pathological Brain Oscillations With Neurofeedback: A Systems Neuroscience Framework.” Frontiers in Human Neuroscience 8: 1008. 10.3389/fnhum.2014.01008. 25566028 PMC4270171

[jsr70232-bib-0044] Sanchez‐Vives, M. V. 2020. “Origin and Dynamics of Cortical Slow Oscillations.” Current Opinion in Physiology 15: 217–223. 10.1016/j.cophys.2020.04.005.

[jsr70232-bib-0045] Schmidt‐Nielsen, K. 1984. Scaling, Why Is Animal Size So Important. Cambrdige University Press.

[jsr70232-bib-0046] Shannon, C. E. 1948. “A Mathematical Theory of Communication.” Bell System Technical Journal 27, no. 3: 379–423. 10.1002/j.1538-7305.1948.tb01338.x.

[jsr70232-bib-0047] Shew, W. L. , and D. Plenz . 2013. “The Functional Benefits of Criticality in the Cortex.” Neuroscientist: A Review Journal Bringing Neurobiology, Neurology and Psychiatry 19, no. 1: 88–100. 10.1177/1073858412445487. 22627091

[jsr70232-bib-0048] Sriraam, N. , T. K. Padma Shri , and U. Maheshwari . 2016. “Recognition of Wake‐Sleep Stage 1 Multichannel Eeg Patterns Using Spectral Entropy Features for Drowsiness Detection.” Australasian Physical & Engineering Sciences in Medicine 39, no. 3: 797–806. 10.1007/s13246-016-0472-8. 27550443

[jsr70232-bib-0049] Touil, M. , L. Bahatti , and A. E. Magri . 2022. “Sleep's Depth Detection Using Electroencephalogram Signal Processing and Neural Network Classification.” Journal of Medical Artificial Intelligence 5: 9. 10.21037/jmai-22-32.

[jsr70232-bib-0050] Van Essen, D. C. , C. J. Donahue , T. S. Coalson , H. Kennedy , T. Hayashi , and M. F. Glasser . 2019. “Cerebral Cortical Folding, Parcellation, and Connectivity in Humans, Nonhuman Primates, and Mice.” Proceedings of the National Academy of Sciences 116, no. 52: 26173–26180. 10.1073/pnas.1902299116. PMC6936571 31871175

[jsr70232-bib-0051] Xu, Y. , A. Schneider , R. Wessel , and K. B. Hengen . 2024. “Sleep Restores an Optimal Computational Regime in Cortical Networks.” Nature Neuroscience 27, no. 2: 328–338. 10.1038/s41593-023-01536-9. 38182837 PMC11272063

[jsr70232-bib-0052] Zaccarelli, N. , B.‐L. Li , I. Petrosillo , and G. Zurlini . 2013. “Order and Disorder in Ecological Time‐Series: Introducing Normalized Spectral Entropy.” Ecological Indicators 28: 22–30. 10.1016/j.ecolind.2011.07.008.

[jsr70232-bib-0053] Zimmern, V. 2020. “Why Brain Criticality Is Clinically Relevant: A Scoping Review.” Frontiers in Neural Circuits 14: 54. 10.3389/fncir.2020.00054. 32982698 PMC7479292

