# Supplementary material for: Sleep Deprivation in Mice: Looking Beyond the Slow Wave Rebound
Source: J Sleep Res. 2025 Nov 8;35(3):e70232. doi: 10.1111/jsr.70232 (PMC13193521; doi:10.1111/jsr.70232)
Supplement: Supplementary file 1 — Data S1: jsr70232‐sup‐0001‐supinfo.docx. [file JSR-35-e70232-s001.docx]

**Supplementary analysis: preREM-REM-postREM triplet**

**Introduction**

The architecture of sleep is widely recognized as critical for memory consolidation, with distinct contributions arising from non-rapid eye movement (NREM) and rapid eye movement (REM) sleep stages. Traditionally, models have emphasized the role of NREM sleep in the reactivation and stabilization of hippocampal-dependent memories through mechanisms like sleep spindles and sharp-wave ripples (Klinzing et al., 2019). REM sleep, by contrast, has been implicated in integrating emotional salience and promoting synaptic plasticity, fostering abstraction and generalization (Stickgold & Walker, 2013). However, emerging work suggests that sleep should not be viewed as disjointed stages but rather as structured sequences, specifically NREM–REM–NREM triplets that together form a dynamic system facilitating memory encoding, recalibration, and consolidation (Lendner et al., 2023).

During REM sleep, desynchronization between brain regions such as the medial temporal lobe and prefrontal cortex appears to reset accumulated synaptic weights, thereby preparing the brain for continued processing in the subsequent NREM phase (Lendner et al., 2023; Miyawaki & Mizuseki, 2022). In rodents, hippocampal theta rhythms during REM and the reemergence of ripple activity in the following NREM period have been linked to improved recall and spatial navigation (Hayat et al., 2022). This cyclical recalibration allows for adaptive memory prioritization, maintaining neural flexibility while preventing over-consolidation or interference. As a result, the NREM–REM–NREM triplet may be considered a neurophysiological unit optimized for efficient learning and synaptic homeostasis.

However, in rodents, sleep is naturally more fragmented, and the concept of a continuous NREM–REM–NREM sequence requires adaptation. Instead, rodent studies operationalize this process as a preREM-REM-postREM triplet, where shorter and more variable sleep episodes still preserve the functional organization required for memory processing (Lendner et al., 2023; Miyawaki & Mizuseki, 2022). In these triplets, REM sleep continues to serve as a physiological "reset" point, disrupting cortical synchrony to prevent synaptic overpotentiation and promoting theta-driven processing in the hippocampus. This is followed by postREM periods where recalibrated networks can support further consolidation. Thus, sleep-dependent memory processing emerges not from isolated stages, but from coordinated triplets that exploit the distinct yet complementary functions of NREM and REM sleep.

**Methods**

We replicated the triplet detection established by Lendner et al. (2023). Specifically, we identified REM episodes lasting at least 30 seconds in duration and extracted the immediately preceding and following segments, which were time-normalized to the duration of the corresponding REM episode. To maintain the integrity of this triplet structure, we implemented an inclusion criteria: any REM episode that occurred at the beginning or end of a recording such that either a pre- or postREM segment could not be fully captured was excluded from analysis. Power spectra were computed using the Welch method (n = 2000 point symmetric Hann window, 4 sec, 50% overlap) and the default parametrization of the FOOOF procedure were used to extract the spectral slopes.

A three-way repeated-measures ANOVA was conducted with DAYS (6 levels: Baseline, Sleep Restriction 1, Sleep Restriction 3, Sleep Restriction 5, Recovery 1, Recovery 3), REGION (2 levels: frontal, parietal), and TRIPLET (3 levels: preREM, REM, postREM) as within-subject factors. In this design, TRIPLET was nested within REGION, and REGION was in turn nested within DAY**S**, reflecting the hierarchical structure of the data and preserving temporal and anatomical dependencies. The analysis tested for main effects and interactions between these factors to evaluate how the spectral slope varied across experimental days, cortical regions, and temporal segments within sleep cycles. The ANOVA was implemented using TIBCO Statistica (version 14.0.1.25), assuming normally distributed residuals and sphericity, with Greenhouse-Geisser corrections applied when violations occurred. Post hoc comparisons were Bonferroni-adjusted. Statistical significance was considered α > 0.01.

**Results & Discussion**

Descriptive statistics are reported in Table S1-7. Inferencial statistics are reported in Table S8. There was a highly significant main effect of TRIPLET (Graph S1; F(2, 14) = 169.55, p < .001, η²ₚ = .96), indicating strong modulation across time segments and the main effect of DAYS remained statistically significant under the stricter threshold (Graph S1-2; F(5, 35) = 5.29, p = .001, η²ₚ = .43.). Post hoc comparisons using Bonferroni correction revealed the following for the TRIPLET main effect: preREM vs. REM (Mean difference = 0.49, SE = 0.03, p < .001); preREM vs. postREM (Mean difference = 0.55, SE = 0.03, p < .001); REM vs. postREM (Mean difference = 0.05, SE = 0.03, p = .415) and for the DAYS main effect: a significant difference was observed between Baseline and Sleep Restriction 1 (Mean difference = –0.16, SE = 0.05, p = .042). Additionally, Sleep Restriction 1 significantly differed from Recovery 1 (Mean difference = 0.22, SE = 0.05, p = .001) and from Recovery 3 (Mean difference = 0.17, SE = 0.05, p = .018).

Therefore, the present data failed to replicate some of the findings of Lendner and his colleges (2023), namely, that REM is characterised by the deepest sleep as indexed by the spectral slope neither on frontal nor on parietal regions. However, we did find a modulatiory effect of REM segments on postREM. That is, preREM segments are characterised by the steepest spectra, followed by the REM and then the postREM segments. This, however, cannot be causally attributed to REM sleep due to the composition of the preREM and postREM periods (Graph S4). Namely, while during preREM mainly NREM dominates, postREM segments are highly fragmented by wakefulness resulting in flat power spectra.

Graph S1. Visual depiction of the spectral slope throughout the time-normalized preREM, REM, postREM triplets.


*Note*: Error bars denote standard errors.

Graph S2. Visual depiction of the spectral slope throughout the experimental days and averaged over the time-normalized preREM, REM, postREM triplets.


*Note*: Error bars denote standard errors; BL: Baseline, SR1-5: Sleep Restriction 1-5, R1-3: Recovery 1-3.

Graph S3. Visual depiction of the spectral slope throughout the experimental days and over the time-normalized preREM, REM, postREM triplets.


*Note*: Error bars denote standard errors; BL = Baseline, SR = Sleep Restriction, R = Recovery. Note that the interaction of the TRIPLET and DAYS did not reach significance, this graph is presented for enhanced visualization.

Graph S4. Composition of preREM and postREM segments.


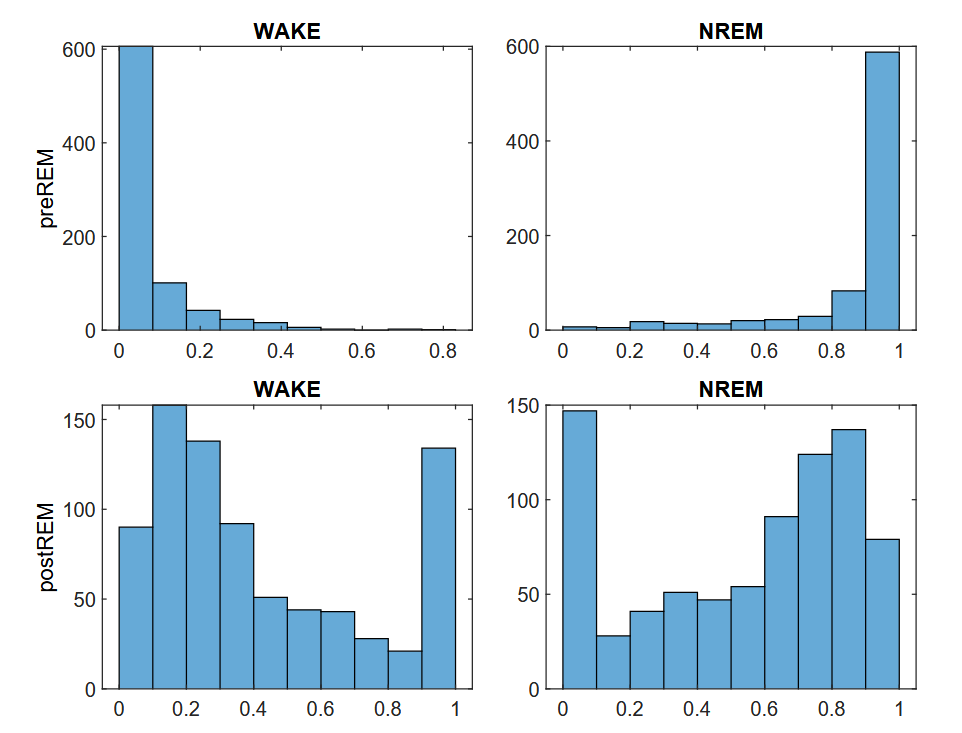

*Note*: Horizontal axis denote percentages (0-1 translates to 0%-100%), while vertixal axis refer to frequencies.

Table S1. Descriptive statistics of the spectral slope throughout the experimental days.

| DAYS | Mean | SD |
| --- | --- | --- |
| 1 | 3.452246 | 0.040062 |
| 2 | 3.608014 | 0.061654 |
| 3 | 3.518317 | 0.068755 |
| 4 | 3.525819 | 0.095547 |
| 5 | 3.386131 | 0.094226 |
| 6 | 3.437269 | 0.062737 |

Note: DAYS 1: Baseline; DAYS 2: Sleep Restriction 1; DAYS 3: Sleep Restriction 3; DAYS 4: Sleep Restriction 5; DAYS 5: Recovery 1; DAYS 6: Recovery 3.

Table S2. Descriptive statistics of the spectral slope throughout cortical regions.

| REGION | Mean | SD |
| --- | --- | --- |
| 1 | 3.418957 | 0.071408 |
| 2 | 3.556975 | 0.088336 |

Note: REGION 1: frontal; REGION 2: parietal.

Table S3. Descriptive statistics of the spectral slope throughout time-normalized preREM, REM, postREM triplets.

| TRIPLET | Mean | SD |
| --- | --- | --- |
| 1 | 3.834474 | 0.056333 |
| 2 | 3.340430 | 0.080730 |
| 3 | 3.288994 | 0.067009 |

Note: TRIPLET 1: preREM; TRIPLET 2: REM; TRIPLET 3: postREM

Table S4. Descriptive statistics of the spectral slope throughout the interaction between the experimental days and cortical regions.

| DAYS | REGION | Mean | SD |
| --- | --- | --- | --- |
| 1 | 1 | 3.372870 | 0.037764 |
| 1 | 2 | 3.531623 | 0.068695 |
| 2 | 1 | 3.529982 | 0.085783 |
| 2 | 2 | 3.686046 | 0.072236 |
| 3 | 1 | 3.437880 | 0.080366 |
| 3 | 2 | 3.598753 | 0.087586 |
| 4 | 1 | 3.441732 | 0.103212 |
| 4 | 2 | 3.609907 | 0.117089 |
| 5 | 1 | 3.361534 | 0.103030 |
| 5 | 2 | 3.410727 | 0.114026 |
| 6 | 1 | 3.369743 | 0.056208 |
| 6 | 2 | 3.504796 | 0.091816 |

Note: DAYS 1: Baseline; DAYS 2: Sleep Restriction 1; DAYS 3: Sleep Restriction 3; DAYS 4: Sleep Restriction 5; DAYS 5: Recovery 1; DAYS 6: Recovery 3. REGION 1: frontal; REGION 2: parietal.

Table S5. Descriptive statistics of the spectral slope throughout the interaction between the experimental days and triplets.

| DAYS | TRIPLET | Mean | SD |
| --- | --- | --- | --- |
| 1 | 1 | 3.818786 | 0.030794 |
| 1 | 2 | 3.320516 | 0.067680 |
| 1 | 3 | 3.217437 | 0.046315 |
| 2 | 1 | 3.938030 | 0.062325 |
| 2 | 2 | 3.430529 | 0.073140 |
| 2 | 3 | 3.455483 | 0.073935 |
| 3 | 1 | 3.893332 | 0.064401 |
| 3 | 2 | 3.378871 | 0.085969 |
| 3 | 3 | 3.282748 | 0.065294 |
| 4 | 1 | 3.880933 | 0.072944 |
| 4 | 2 | 3.393286 | 0.100124 |
| 4 | 3 | 3.303238 | 0.124552 |
| 5 | 1 | 3.738576 | 0.073025 |
| 5 | 2 | 3.236241 | 0.106999 |
| 5 | 3 | 3.183574 | 0.109279 |
| 6 | 1 | 3.737188 | 0.066227 |
| 6 | 2 | 3.283138 | 0.074860 |
| 6 | 3 | 3.291483 | 0.068286 |

Note: DAYS 1: Baseline; DAYS 2: Sleep Restriction 1; DAYS 3: Sleep Restriction 3; DAYS 4: Sleep Restriction 5; DAYS 5: Recovery 1; DAYS 6: Recovery 3. TRIPLET 1: preREM; TRIPLET 2: REM; TRIPLET 3: postREM.

Table S6. Descriptive statistics of the spectral slope throughout the interaction between the cortical regions and triplets.

| REGION | TRIPLET | Mean | SD |
| --- | --- | --- | --- |
| 1 | 1 | 3.762001 | 0.067101 |
| 1 | 2 | 3.275513 | 0.074301 |
| 1 | 3 | 3.219356 | 0.076659 |
| 2 | 1 | 3.906947 | 0.064513 |
| 2 | 2 | 3.405347 | 0.122492 |
| 2 | 3 | 3.358631 | 0.084221 |

Note: REGION 1: frontal; REGION 2: parietal. TRIPLET 1: preREM; TRIPLET 2: REM; TRIPLET 3: postREM.

Table S7. Descriptive statistics of the spectral slope throughout the interaction between the experimental days, cortical regions and triplets.

| DAYS | REGION | TRIPLET | Mean | SD |
| --- | --- | --- | --- | --- |
| 1 | 1 | 1 | 3.724978 | 0.037353 |
| 1 | 1 | 2 | 3.261987 | 0.047123 |
| 1 | 1 | 3 | 3.131645 | 0.056163 |
| 1 | 2 | 1 | 3.912594 | 0.046656 |
| 1 | 2 | 2 | 3.379046 | 0.104803 |
| 1 | 2 | 3 | 3.303229 | 0.071982 |
| 2 | 1 | 1 | 3.858395 | 0.086256 |
| 2 | 1 | 2 | 3.358558 | 0.087412 |
| 2 | 1 | 3 | 3.372994 | 0.098744 |
| 2 | 2 | 1 | 4.017666 | 0.058899 |
| 2 | 2 | 2 | 3.502499 | 0.113175 |
| 2 | 2 | 3 | 3.537971 | 0.072957 |
| 3 | 1 | 1 | 3.815502 | 0.078469 |
| 3 | 1 | 2 | 3.285168 | 0.096812 |
| 3 | 1 | 3 | 3.212971 | 0.075769 |
| 3 | 2 | 1 | 3.971162 | 0.067317 |
| 3 | 2 | 2 | 3.472574 | 0.118983 |
| 3 | 2 | 3 | 3.352524 | 0.084561 |
| 4 | 1 | 1 | 3.800893 | 0.093375 |
| 4 | 1 | 2 | 3.290928 | 0.099569 |
| 4 | 1 | 3 | 3.233374 | 0.128641 |
| 4 | 2 | 1 | 3.960973 | 0.080717 |
| 4 | 2 | 2 | 3.495644 | 0.143713 |
| 4 | 2 | 3 | 3.373102 | 0.139345 |
| 5 | 1 | 1 | 3.701533 | 0.090242 |
| 5 | 1 | 2 | 3.224984 | 0.103671 |
| 5 | 1 | 3 | 3.158086 | 0.118457 |
| 5 | 2 | 1 | 3.775620 | 0.084469 |
| 5 | 2 | 2 | 3.247498 | 0.143665 |
| 5 | 2 | 3 | 3.209063 | 0.124402 |
| 6 | 1 | 1 | 3.670706 | 0.062346 |
| 6 | 1 | 2 | 3.231453 | 0.058417 |
| 6 | 1 | 3 | 3.207068 | 0.065806 |
| 6 | 2 | 1 | 3.803669 | 0.080617 |
| 6 | 2 | 2 | 3.334822 | 0.125828 |
| 6 | 2 | 3 | 3.375897 | 0.094261 |

Note: DAYS 1: Baseline; DAYS 2: Sleep Restriction 1; DAYS 3: Sleep Restriction 3; DAYS 4: Sleep Restriction 5; DAYS 5: Recovery 1; DAYS 6: Recovery 3. REGION 1: frontal; REGION 2: parietal. TRIPLET 1: preREM; TRIPLET 2: REM; TRIPLET 3: postREM.

Table S8. Repeated Measures Analysis of Variance with Effect Sizes; Sigma-restricted parameterization; Effective hypothesis decomposition.

| Effect | DF | F | p | Partial eta-squared |
| --- | --- | --- | --- | --- |
| Intercept | 1 | 2783.773 | 0.000000 | 0.997492 |
| Error | 7 |  |  |  |
| DAYS | 5 | 5.291 | 0.001008 | 0.430497 |
| Error | 35 |  |  |  |
| REGION | 1 | 2.289 | 0.174087 | 0.246390 |
| Error | 7 |  |  |  |
| TRIPLET | 2 | 169.555 | 0.000000 | 0.960352 |
| Error | 14 |  |  |  |
| DAYS*REGION | 5 | 1.529 | 0.205957 | 0.179313 |
| Error | 35 |  |  |  |
| DAYS*TRIPLET | 10 | 1.376 | 0.209452 | 0.164293 |
| Error | 70 |  |  |  |
| REGION*TRIPLET | 2 | 0.044 | 0.956702 | 0.006303 |
| Error | 14 |  |  |  |
| DAYS*REGION*TRIPLET | 10 | 2.356 | 0.018312 | 0.251834 |
| Error | 70 |  |  |  |

**References**

Hayat, H., Marmelshtein, A., Krom, A. J., Sela, Y., Tankus, A., Strauss, I., Fahoum, F., Fried, I., & Nir, Y. (2022). Reduced neural feedback signaling despite robust neuron and gamma auditory responses during human sleep. *Nature Neuroscience*, *25*(7), 935–943. https://doi.org/10.1038/s41593-022-01107-4

Klinzing, J. G., Niethard, N., & Born, J. (2019). Mechanisms of systems memory consolidation during sleep. *Nature Neuroscience*, *22*(10), 1598–1610. https://doi.org/10.1038/s41593-019-0467-3

Lendner, J. D., Niethard, N., Mander, B. A., van Schalkwijk, F. J., Schuh-Hofer, S., Schmidt, H., Knight, R. T., Born, J., Walker, M. P., Lin, J. J., & Helfrich, R. F. (2023). Human REM sleep recalibrates neural activity in support of memory formation. *Science Advances*, *9*(34), eadj1895. https://doi.org/10.1126/sciadv.adj1895

Miyawaki, H., & Mizuseki, K. (2022). De novo inter-regional coactivations of preconfigured local ensembles support memory. *Nature Communications*, *13*(1), 1272. https://doi.org/10.1038/s41467-022-28929-x

Stickgold, R., & Walker, M. P. (2013). Sleep-dependent memory triage: Evolving generalization through selective processing. *Nature Neuroscience*, *16*(2), 139–145. https://doi.org/10.1038/nn.3303
